# Self-adaptive dual-strategy differential evolution algorithm

**DOI:** 10.1371/journal.pone.0222706

**Published:** 2019-10-03

**Authors:** Meijun Duan, Hongyu Yang, Shangping Wang, Yu Liu

**Affiliations:** 1 National Key Laboratory of Fundamental Science on Synthetic Vision, Sichuan University, Chengdu, China; 2 College of Computer Science, Sichuan University, Chengdu, China; 3 Science and Technology on Electronic Information Control Laboratory, Chengdu, China; Universiti Sains Malaysia, MALAYSIA

## Abstract

Exploration and exploitation are contradictory in differential evolution (DE) algorithm. In order to balance the search behavior between exploitation and exploration better, a novel self-adaptive dual-strategy differential evolution algorithm (SaDSDE) is proposed. Firstly, a dual-strategy mutation operator is presented based on the “DE/best/2” mutation operator with better global exploration ability and “DE/rand/2” mutation operator with stronger local exploitation ability. Secondly, the scaling factor self-adaption strategy is proposed in an individual-dependent and fitness-dependent way without extra parameters. Thirdly, the exploration ability control factor is introduced to adjust the global exploration ability dynamically in the evolution process. In order to verify and analyze the performance of SaDSDE, we compare SaDSDE with 7 state-of-art DE variants and 3 non-DE based algorithms by using 30 Benchmark test functions of 30-dimensions and 100-dimensions, respectively. The experiments results demonstrate that SaDSDE could improve global optimization performance remarkably. Moreover, the performance superiority of SaDSDE becomes more significant with the increase of the problems’ dimension.

## Introduction

DE is a stochastic optimization method based on population for real-parameter optimization problems, which is proposed by Storn and Price [1]. As a kind of heuristic global search evolution algorithm, differential evolution (DE) has an evolution mechanism similar to the other evolution algorithms, all of which are mutated, crossed and selected. Unlike that the other evolution algorithms (EAs) use the defined probability distribution function, the DE selects individuals from the current population to do differential operations and multiplies by scaling factor. It currently is a very attractive evolutionary algorithm for optimization in continuous search spaces, mainly for its simplicity, a small number of parameters to tune and notable performance. At present, DE is widely used in the research and engineering fields, such as classification [2], neural network training [3–4], data clustering [5], solar energy [6].

Exploration and exploitation are contradictory for most EAs [7]. To further improve the exploitation and exploration capability of DE algorithm, a novel self-adaptive dual-strategy differential evolution algorithm (SaDSDE) is proposed. In summary, the main contributions of this paper are as follows. (1) A dual-strategy mutation operator is proposed. In the proposed mutation operator, the exploitation and exploration capability of mutation operator are simultaneously considered. (2) The scaling factor self-adaption strategy is proposed in an individual-dependent and fitness-dependent way. (3) A dynamic adjustment strategy of exploration ability control factor is introduced to adjust the exploration ability of DE in different stages of evolution process. (4) The performance of SaDSDE is verified by comparing it with 7 existing state-of-art DE variants and 3 non-DE algorithms on 30 benchmark functions. The experimental results demonstrate the effectiveness of the proposed SaDSDE. The performance sensitivities to population size, crossover probability and the impacts of the proposed algorithmic components are also investigated.

The rest of this paper is organized as follows. Section 2 briefly introduces the original DE algorithm and reviews the related works on the DE variants. In Section 3, the proposed SaDSDE algorithm is presented in detail. The experiment comparisons and analysis with 7 state-of-art algorithms and 3 non-DE algorithms are given in Section 4. Finally, Section 5 draws the conclusions of this work, and future work is also described in this section.

## Related work

### 2.1 Original differential evolution algorithm

DE is based on population evolution. Generally, DE is composed of four components: initialization, mutation, crossover and selection. After initialization, DE enters a loop of mutation, crossover and selection. The details of main components are introduced as follows. Without loss of generality, this paper considers minimization problems. For *D*-dimensional minimum optimization problems:

$$\left\{ \begin{array}{l} \text{min}f(x_{1},x_{2}\ldots ,x_{D})\\ s.t\;\;\;x_{j}^{\text{min}}\leq x_{i,j}\leq x_{j}^{\text{max}},\;\;\;\;j=1,2,\ldots .,D \end{array}\right.$$ (1)

where *i* = {1, 2, ⋯, *N*_*p*_}, *N*_*p*_ is the population size, $x_{j}^{\text{min}}$ and $x_{j}^{\text{max}}$ are respectively the lower bound and upper bound of *x*_*j*_.

#### 2.1.1 Mutation

It is mainly to generate mutation vector by scaling the difference of different individuals. There are many mutation operators proposed by Storn and Price [8, 9] and a widely used mutation strategy is listed as follows:

$$V_{i}^{t}=X_{r1}^{t}+F\cdot (X_{r2}^{t}-X_{r3}^{t})$$ (2)

where *t* indicates the generation, the indexes *r*_1_ ~ *r*_3_ are distinct integers randomly chosen from the set {1, 2, ⋯, *N*_*p*_}\{*i*}, $V_{i}^{t}$ is the mutation vector of the *i*th target vector $X_{i}^{t}$. The scaling factor *F* controls the amplification of the difference vector and is closely related to convergence speed. The small scaling factor determines the exploitation ability. On the contrary, the large scaling factor determines the exploration ability. It is note that the optimization performance of DE mainly depends on the choice of mutation strategies and the setting of control parameters.

#### 2.1.2 Crossover

By crossover operation on the mutation vector $V_{i}^{t}$ and the *i*th target vector $X_{i}^{t}$, the trial vector is generated. The diversity of the population can be increased by crossover operation. There are mainly three classic crossover operators: binomial crossover, exponential crossover and rotationally invariant arithmetic crossover operators. The following binomial crossover operator is the most commonly used.

$$U_{i,j}^{t}=\left\{ \begin{array}{l} V_{i,j}^{t}\;\;\;if\;rand_{j}\leq CR\;\;or\;\;j=j_{rand}\\ X_{i,j}^{t}\;\;\;otherwise\;\; \end{array}\right.\;\;\;$$ (3)

where *rand*_*j*_ is a uniformly distributed random variable within (0, 1). *j*_*rand*_ is randomly chosen from the set {1, 2,⋯, *D*}, which can prevents a direct copy from $X_{i}^{t}$ to $U_{i}^{t}$. The crossover probability *CR* is a user-defined crossover factor restricted to (0, 1], which controls the diversity of the population and is closely connected with exploration power.

#### 2.1.3 Selection

After crossover, the objective function value of the trial vector can be obtained according to the optimization problem. The newly generated trial vectors are evaluated and compared with the target vectors. A greedy strategy is adopted to perform the selection operation in DE. The superior of the target vector $X_{i}^{t}$ and trial vector $U_{i}^{t}$ will survive in the next generation. In mathematics, one has

$$X_{i}^{t+1}=\left\{ \begin{array}{l} U_{i}^{t}\;\;f(U_{i}^{t})\leq f(X_{i}^{t})\\ X_{i}^{t}\;\;others \end{array}\right.$$ (4)

#### 2.2 Literature review

The performance of DE is mainly influenced by mutation mode, control parameters (i.e. population size (*N*_*p*_), scaling factor (*F*) and crossover probability (*CR*)) and crossover mode. Inappropriate configurations of mutation strategies and control parameters can cause stagnation or premature convergence. Therefore, many scholars have proposed a series of improved DE algorithms [10–46].

To avoid manually tuning parameters, researchers have developed some techniques to automatically set the parameter values. Ryoji and Alex [10] used a historical memory of successful control parameters to guide the selection of future control parameters and proposed a parameter adaptation technique for DE (SHADE). Rcr-JADE [11] was an improved version of JADE [12] which employs successful parameters to repair crossover rate. To enhance the performance of L-SHADE algorithm, Awad and Ali et al. [13] proposed LSHADE-EpSin by using an adaptive approach based on sinusoidal formulas to adapt the scaling factor. In addition, some scholars have proposed adaptive strategies for the population size. Zhu et al. [14] proposed an adaptive population tuning scheme. In SapsDE [15], a self-adaptive population resizing mechanism was employed to adjust the population size. Chen and Zhao [16] et al. proposed population adaptive differential evolution (PADE). Award and Ali [17] et al. presented ensemble sinusoidal differential evolution with niching-based population reduction (called EsDEr-NR).

A large number of studies have carried out on improving mutation strategies. A part of studies focused on single-mutation mode. In JADE [12], an optional external archive is combined with a mutation strategy DE/current-to-pbest/1 that utilizes historical information to direct population searching. Wang et al. [18] proposed a self-adaptive differential evolution algorithm with improved mutation mode (IMMSADE) by improving “DE/rand/1”. Cai et al. [19] designed a neighborhood-dependent directional mutation operator and presented a neighborhood-adaptive DE (NaDE). A novel “DE/current-to-SP-best-ring/1” mutation operation is introduced in decentralizing and coevolving differential evolution (DCDE), proposed by Tang [20]. A novel and effective adaptation scheme is used to update the crossover rate in adaptive guided differential evolution algorithm (AGDE) [21]. He and Zhou [22] presented a novel DE variant with covariance matrix self-adaptation (DECMSA). In EFADE [23], a new triangular mutation operator is introduced. Cai et al. [24] presented an adaptive social learning (ASL) strategy to extract the neighborhood relationship information. The best search strategies and parameters of an EA are generally different in solving different optimization problems. Therefore, in order to improve the performance of an EA like DE, researchers make efforts to realize an ensemble of multiple strategies and parameters [25–33]. Qin et al. [25] proposed a self-adaptive DE algorithm (SaDE), both trial vector generation strategies and their associated control parameter values were gradually self-adapted. Wang et al. [26] introduced a Composite Differential Evolution algorithm (CoDE), which used three trial vector generation strategies and three control parameter settings. Mallipeddi et al. [27] employed an ensemble of mutation strategies and control parameters with the DE (EPSDE). Elsayed et al. [28] used multiple search operators in conjunction with multiple constraint handing techniques. Wu and Mallipeddi et al. [29] proposed a multi-population ensemble DE (MPEDE). YEH et al. [30] mixed Gauss mutation and the “DE/best/1” operator. Cui et al. [31] proposed an adaptive multiple-elites-guided composite differential evolution algorithm with a shift mechanism (AMECoDEs). Wu et al. [32] focused on the high-level ensemble of different DE variants and proposed a new algorithm named EDEV. Lin and Ma et al. [33] proposed an adaptive immune-inspired multi-objective algorithm (AIMA).

In addition to the modification of mutation and control parameters optimization, enhancements in crossover operators have also been investigated, such as covariance matrix learning operator [34], hybrid linkage crossover [35], a crossover operator utilizing eigenvectors of covariance matrix of individual solutions [36], superior-inferior crossover scheme [37], an adaptive hybrid crossover operator(AHX) [38], optional blending crossover scheme [39] and a multiple exponential recombination that inherits all the main advantages of existing crossover operators [40].

Through the synergistic mechanism, a hybrid algorithm could take advantage of various merits within different algorithms, and then yields more favorable performance than a single algorithm. Some preliminary research manifest that hybrid optimizers are effective and competent for global optimization. Li et al. [41] proposed a new hybrid algorithm, called as differential evolution algorithm (DE) / artificial bee colony (ABC) algorithm. Vaisakh et al. [42] came up with a hybrid approach involving differential evolution (DE) and bacterial foraging optimization algorithm (BFOA). Ponsich and Coello [43] hybridized DE with Tabu Search (TS). Gu et al. [44] mixed binary differential evolution (BDE) and Tabu search (TS) to propose the memetic algorithm. Le et al. [45] merged differential evolution and harmony search. Nenavath and Jatoth [46] hybridized sine cosine algorithm with differential evolution.

## SaDSDE algorithm

### 3.1 Dual-strategy mutation operator

In the original mutation strategies [8, 9], “DE/best/1” and “DE/best/2” conduct mutation on the best individual with better local exploitation ability and fast convergence speed, but they are easy to suffer premature convergence. “DE/rand/1” and “DE/rand/2” do mutation based on random individuals with stronger global exploration ability, but they are lack of the guidance of the optimal individual, so the convergence speed is slow. “DE/current-to-best/1” performs mutation based on the parent individual and the optimal individual with high convergence precision, but it is easy to fall into local optimum. Above mutation strategies are either too greedy or too stochastic. Therefore, in order to balance the exploitation and exploration better, a novel dual-strategy mutation operator is proposed based on “DE/best/2” and “DE/rand/2”, which is shown in Eqs (5)–(7).

$$V_{i\_1}^{t}=X_{b}^{t}+F_{i}^{t}\cdot (X_{r1}^{t}-X_{r2}^{t})+F_{i}^{t}\cdot (X_{r3}^{t}-X_{r4}^{t})$$ (5)

$$V_{i\_2}^{t}=X_{r1}^{t}+F_{i}^{t}\cdot (X_{r2}^{t}-X_{r3}^{t})+F_{i}^{t}\cdot (X_{r4}^{t}-X_{r5}^{t})$$ (6)

$$V_{i}^{t}=\omega_{i\_1}\cdot V_{i\_1}^{t}+\lambda \cdot \omega_{i\_2}\cdot V_{i\_2}^{t}$$ (7)

where $X_{b}^{t}$ is the best solution at the current generation *t*. *r*_1_ ≠ *r*_2_ ≠ *r*_3_ ≠ *r*_4_ ≠ *r*_5_ are randomly chosen from the set {1, ⋯, *N*_*p*_}\{*i*}, $F_{i}^{t}$ is the scaling factor of the *i*th individual. *ω*_*i*_1_ and *ω*_*i*_2_ are the weights of two mutation operators, which take the value of either 0 or 1. If *rand*_*i*_ () < 0.5, *ω*_*i*_1_ = 1, *ω*_*i*_2_ = 0; otherwise, *ω*_*i*_1_ = 0, *ω*_*i*_2_ = 1. Two weights satisfy *ω*_*i*_1_ + *ω*_*i*_2_ = 1. In the above mutation scheme, only one of the two strategies is used for each vector depending on a uniformly distributed random value within the range (0, 1). *λ* is the exploration ability control factor and adjusts the exploration ability of mutation operator.

### 3.2 The scaling factor self-adaption strategy

The solution quality and the fitness are closely related. For the solution quality, the fitness is the larger the better. Therefore, the scaling factor is tuned according to the fitness in a self-adaptive and individual-dependent way, as is described in Eq (8).

$$F_{i}^{t}=(f_{\text{max}}^{t}-f(X_{i}^{t}))/(f_{\text{max}}^{t}-f_{\text{min}}^{t})$$ (8)

Where $f(X_{i}^{t})$ is the fitness of the individual $X_{i}^{t}$, $f_{\text{max}}^{t}$ is the maximum fitness at the current generation, and $f_{\text{min}}^{t}$ is the minimum fitness. In particular, when $f_{\text{max}}^{t}$ is equal to $f_{\text{min}}^{t}$, $F_{i}^{t}$ is set to a uniformly distributed random variable within (0, 1) to increase disturbance. Superior individuals tend to be assigned with smaller parameter values so as to exploit their neighborhoods in which better solutions, while inferior individuals tend to be assigned with larger parameter values so as to explore further areas in the solution space.

### 3.3 The dynamic adjustment strategy of the exploration ability control factor λ

A key problem in many evolutionary algorithms is premature convergence, especially in the later stage of evolution. However, promoting strong global exploration ability at all stages of an evolutionary process might even be counterproductive in a phase where high exploitation is needed. In general, most algorithms perform more effectively with a high convergence rate in the earlier stage of the searching process relative to the later stage, especially for multimodal functions. At the later stage, the algorithms are easily trapped in local optimal solutions due to poor population diversity. Therefore, the dynamic adjustment strategy of the exploration ability control factor is introduced according to the evolution property, which is shown in Eq (9).

$$\lambda =1-\text{cos}({\left(t/T\right)}^{2})$$ (9)

**Fig 1:**
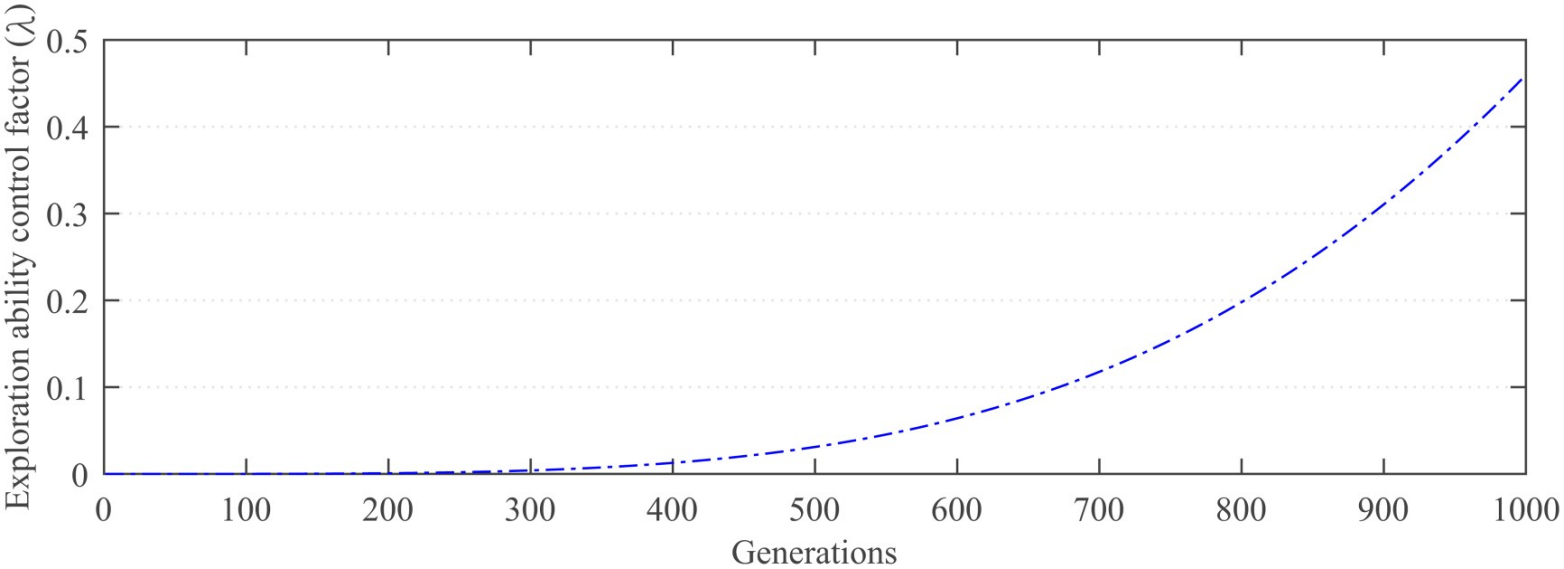
The dynamic adjustment curve of λ.

Taking 1000 generations as an example, the dynamic adjustment curve of the exploration ability control factor is shown in the Fig 1. During evolution, the population diversity is better and *λ* is assigned a smaller value at the earlier stage. In response to the decreased diversity of the population at the later stage of evolution, *λ* is assigned a larger value to increase the proportions of the explorative mutation operator.

Based on all the components described above, the pseudo-code of the complete procedure of SaDSDE is outlined in Algorithm 1. Comparing to the basic DEs, the time complexity of proposed SaDSDE is still *O*(*T* * *N*_*p*_ * *D*) without extra time complexity. Herein, *T*, *N*_*p*_ and *D* are respectively the maximum generation number, the population size and the dimension of problem.

**Algorithm 1**. **Pseudo-Code for the SaDSDE**

**Begin**

#01 *t* = 0;

#02 Randomly initialize an initial population $X^{\left(0\right)}=\{X_{i,j}^{\left(0\right)}|\forall i,i=1,\ldots ,N_{p},\forall j,j=1,\ldots ,D\}$, $X_{i,j}^{\left(0\right)}\in [x_{j}^{\text{min}},x_{j}^{\text{max}}]$, *T* = 1000, *CR* = 0.9;

#03 **For** *t* = 1 to *T* **Do**

#04  Calculate the exploration ability control factor *λ* = 1 − cos((*t*/*T*)^2^);

#05  Evaluate $f(X_{i}^{t}|\forall i,i=1,\cdots ,N_{p})$ and find $X_{b}^{t}$;

#06  **For** each individual $X_{i}^{t}$ in current population **Do**

#07   **If $f_{\text{max}}^{t}\neq f_{\text{min}}^{t}$ Then**

#08   Update $F_{i}^{t}=(f_{\text{max}}^{t}-f(X_{i}^{t}))/(f_{\text{max}}^{t}-f_{\text{min}}^{t})$;

#09   **Else**

#10   Set $F_{i}^{t}=rand(0,1)$ to increase disturbance;

#11   **End**

#12   Generate a random real number $r_{i}^{t}=rand(0,1)$;

#13   **If** $r_{i}^{t}<0.5$ **Then**

#14   *ω*_*i*_1_ = 1 and *ω*_*i*_2_ = 0;

#15   **Else**

#16   *ω*_*i*_1_ = 0 and *ω*_*i*_2_ = 1;

#17   **End**

#18   Generate *r*_1_ ≠ *r*_2_ ≠ *r*_3_ ≠ *r*_4_ ≠ *r*_5_ from the set {1,⋯, *N*_*p*_}\{*i*} randomly;

#19    $V_{i\_1}^{t}=X_{b}^{t}+F_{i}^{t}\cdot (X_{r1}^{t}-X_{r2}^{t})+F_{i}^{t}\cdot (X_{r3}^{t}-X_{r4}^{t})$;

#20    $V_{i\_2}^{t}=X_{r1}^{t}+F_{i}^{t}\cdot (X_{r2}^{t}-X_{r3}^{t})+F_{i}^{t}\cdot (X_{r4}^{t}-X_{r5}^{t})$;

#21   **For** *j* = 1 to *D* **Do**

#22    Generate *j*_*rand*_ = *randint*(1, *D*);

#23    **If** *j* = *j*_*rand*_ or *rand*(0,1) < *CR* **Then**

#24     $U_{i,j}^{t}=\omega_{i\_1}\cdot V_{i\_1,j}^{t}+\lambda \cdot \omega_{i\_2}\cdot V_{i\_2,j}^{t};$

#25    **Else**

#26     $U_{i,j}^{t}=X_{i,j}^{t};$

#27    **End If**

#28   **End For**

#29   **If** $f(U_{i}^{t})\leq f(X_{i}^{t})$ **Then**

#30    $X_{i}^{t+1}=U_{i}^{t}$;

#31   **Else**

#32    $X_{i}^{t+1}=X_{i}^{t}$;

#33   **End If**

#34  **End For**

#35 *t* = *t* + 1

#36 **End While**

**End**

## Experiments analysis

### 4.1 Experiments setup

In our experiments, 30 benchmark test functions from references [47–49] are used to test the performance of SaDSDE, which are listed in Table 1. *f*_*1*_*—f*_*11*_ are unimodal functions, *f*_*12*_*-f*_*30*_ are multimodal functions.

**Table 1:** Benchmark test functions.

Name	Function	Domain
Sphere	$f_{1}(x)=\sum_{i=1}^{D}x_{i}^{2}$	[–100,100]^D^
Schwefel 1.2	$f_{2}(x)={\sum_{i=1}^{D}\left(\sum_{j=1}^{i}x_{j}\right)}^{2}$	[–100,100]^D^
Elliptic	$f_{3}(x)=\sum_{i=1}^{D}{\left(10^{6}\right)}^{\frac{i-1}{N-1}}x_{i}^{2}$	[–100,100]^D^
Schwefel 2.22	$f_{4}(x)=\sum_{i=1}^{D}|x_{i}|+\prod_{i=1}^{D}\left|x_{i}\right|$	[–10,10]^D^
Schwefel 2.21	*f*_5_(*x*) = max{|*x*_*i*_|, 1 ≤ *i* ≤ *D*}	[–100,100]^D^
SumSquares	$f_{6}(x)=\sum_{i=1}^{D}ix_{i}^{2}$	[–1,1]^D^
Tablet	$f_{7}(x)=10^{6}x_{1}^{2}+\sum_{i=2}^{D}x_{i}^{2}$	[–100,100]^D^
Zakharov	$f_{8}(x)=\sum_{i=1}^{D}x_{i}^{2}+{\left(\sum_{i=1}^{D}0.5ix_{i}\right)}^{2}+{\left(\sum_{i=1}^{D}0.5ix_{i}\right)}^{4}$	[–5,10]^D^
Bent Cigar	$f_{9}(x)=x_{1}^{2}+10^{6}\sum_{i=2}^{D}x_{i}^{2}$	[–100,100]^D^
Step	$f_{10}(x)={\sum_{i=1}^{D}\left(|x_{i}+0.5|\right)}^{2}$	[–100,100]^D^
Noise Quartic	$f_{11}(x)=\sum_{i=1}^{D}ix_{i}^{4}+rand[0,1)$	[-1.28,1.28]^D^
Rastrigin	$f_{12}(x)=\sum_{i=1}^{D}\left(x_{t}^{2}-10\text{cos}(2\pi x_{i})+10\right)$	[-5.12,5.12]^D^
Griewank	$f_{13}(x)=\sum_{i=1}^{D}x_{i}^{2}/4000-\prod_{i=1}^{D}\text{cos}(x_{i}/\sqrt{i})+1$	[–600,600]^D^
Scaffer’s F6	$f_{14}(x)=\sum_{i=1}^{D}\left(0.5+\frac{{\text{sin}}^{2}(\sqrt{x_{i}^{2}+x_{i+1}^{2}})-0.5}{{\left(1+0.001(x_{i}^{2}+x_{i+1}^{2})\right)}^{2}}\right)x_{D+1}=x_{1}$	[-0.5,0.5]^D^
Salomon	$f_{15}(x)=1-\text{cos}(2\pi \sqrt{\sum_{i=1}^{D}x_{i}^{2}})+0.1\sqrt{\sum_{i=1}^{D}x_{i}^{2}}$	[–100,100]^D^
Ackley	$f_{16}(x)=20+e-20\text{exp}(-0.2\sqrt{\sum_{i=1}^{D}x_{i}^{2}/D})-\text{exp}(\sum_{i=1}^{D}\text{cos}(2\pi x_{i})/D)$	[–32,32]^D^
Rosenbrock	$f_{17}=\sum_{i=1}^{D-1}\left(100{\left(x_{i}^{2}-x_{i+1}\right)}^{2}+{\left(x_{i}-1\right)}^{2}\right)$	[–100,100]^D^
Scaffer2	$f_{18}(x)={\sum_{i=1}^{D}\left(x_{t}^{2}+x_{i+1}^{2}\right)}^{0.25}(\text{sin}(50{\left(x_{t}^{2}+x_{i+1}^{2}\right)}^{0.1})+1)$	[–100,100]^D^
Modified Schwefel	$\begin{array}{l} f_{19}(x)=418.9829*D-\sum_{i=1}^{D}g(z_{i}),z_{i}=x_{i}+4.20968746227503e+002\\ g(z_{i})=\left\{ \begin{array}{ll} z_{i}\;\text{sin}(|z_{i}{|}^{1/2}) & if|z_{i}|<500\\ (500-\text{mod}(z_{i},500))\text{sin}(\sqrt{\left|500-\text{mod}(|z_{i}|,500)\right|})-\frac{{\left(z_{i}-500\right)}^{2}}{1000D} & if\;z_{i}>500\\ (\text{mod}(z_{i},500)-500)\text{sin}(\sqrt{\left|\text{mod}(|z_{i}|,500)-500\right|})-\frac{{\left(z_{i}-500\right)}^{2}}{1000D} & if\;z_{i}<-500 \end{array}\right. \end{array}$	[–100,100]^D^
HappyCat	$f_{20}(x)=|\sum_{i=1}^{D}x_{i}^{2}-D{|}^{1/4}+(0.5\sum_{i=1}^{D}x_{i}^{2}+\sum_{i=1}^{D}x_{i})/D+0.5$	[–100,100]^D^
HGBat	$f_{21}(x)=|{\left(\sum_{i=1}^{D}x_{i}^{2}\right)}^{2}-{\left(\sum_{i=1}^{D}x_{i}\right)}^{2}{|}^{1/2}+(0.5\sum_{i=1}^{D}x_{i}^{2}+\sum_{i=1}^{D}x_{i})/D+0.5$	[–100,100]^D^
Weierstrass	$f_{22}(x)=\sum_{i=1}^{D}\left(\sum_{k=0}^{k_{\text{max}}}\left[a^{k}\;\text{cos}(2\pi b^{k}(x_{i}+0.5))\right]\right)-D\sum_{k=0}^{k_{\text{max}}}[a^{k}\;\text{cos}(2\pi b^{k}\cdot 0.5)],a=0.5,b=3,k_{\text{max}}=20$	[–100,100]^D^
Katsuura	$f_{23}(x)=\frac{10}{D^{2}}\prod_{i=1}^{D}{\left(1+i\sum_{j=1}^{32}\frac{\left|2^{j}x_{i}-round(2^{j}x_{i})\right|}{2^{j}}\right)}^{\frac{10}{D^{1.2}}}-\frac{10}{D^{2}}$	[–5,5]^D^
E_ScafferF6	*f*_24_(*x*) = *f*_14_(*x*_1_,*x*_2_) + ⋯ + *f*_14_(*x*_*D*−1_, *x*_*D*_) + *f*_14_(*x*_D_, *x*_1_)	[–3,1]^D^
Griewank+Rosenbrock	*f*_25_(*x*) = *f*_13_(*f*_17_(*x*_1_,*x*_2_)) + ⋯ *f*_13_(*f*_17_(*x*_*D*−1_, *x*_*D*_)) + *f*_13_(*f*_17_(*x*_D_, *x*_1_))	[-5.12,5.12]^D^
NCRastrigin	$f_{26}=\sum_{i=1}^{D}\left[y_{i}^{2}-10\text{cos}(2\pi y_{i}+10)\right],y_{i}=\left\{ \begin{array}{l} x_{i},|x_{i}|<0.5\\ round(2x_{i})/2,|x_{i}|\geq 0.5 \end{array}\right.$	[–10,10]^D^
Apline	$f_{27}=\sum_{i=1}^{D}\left|x_{i}\;\text{sin}x_{i}+0.1x_{i}\right|$	[–100,100]^D^
Bohachevsky_2	$f_{28}=\sum_{i=1}^{D-1}\left[x_{i}^{2}+2x_{i+1}^{2}-0.3\text{cos}(3\pi x_{i})\text{cos}(3\pi x_{i+1})+0.3\right]$	[–100,100]^D^
Levy and Montalvo 1	$f_{29}(x)=\frac{\pi }{D}(10{\text{sin}}^{2}(\pi y_{1})+\sum_{i=1}^{D-1}{\left(y_{i}-1\right)}^{2}(1+10{\text{sin}}^{2}(\pi y_{i+1}))+{\left(y_{D}-1\right)}^{2}),y=1+\frac{1}{4}(x_{i}+1)$	[–10, 10]^D^
Levy and Montalvo 2	$f_{30}(x)=0.1(10{\text{sin}}^{2}(3\pi x_{1})+\sum_{i=1}^{D-1}{\left(x_{i}-1\right)}^{2}(1+{\text{sin}}^{2}(3\pi x_{i+1})+{\left(x_{D}-1\right)}^{2}(1+{\text{sin}}^{2}(2\pi x_{D})))$	[–5, 5]^D^

### 4.2 Time complexity

The time complexity is calculated as described in [47]. The codes are implemented in Matlab 2015a and run on a PC with an Intel (R) Core (TM) i5-6500 CPU (3.20GHz) and 8GB RAM. The algorithm complexity is listed in Table 2. In Table 2, *T*_0_ denotes the running time of the following program:

$$\begin{array}{l} for\;i=1:1000000\\ x=(double)5.55;\\ x=x+x;x=x./2;x=x*x;x=sqrt(x);x=\text{ln}(x);x=\text{exp}(x);y=x/x;\\ end \end{array}$$

**Table 2:** Time complexity (time in seconds).

Dimensions	*T*_0_	*T*_1_	${\hat{T}}_{2}$	$({\hat{T}}_{2}-T_{1})/T_{0}$
***D* = 30**	0.1160	0.1030	3.6340	30.4394
***D* = 100**		0.1170	3.6175	30.1767

*T*_1_ is the computing time just for Function 3 (Elliptic function) for 200,000 evaluations at a certain dimension *D*. *T*_2_ is the complete computing time for the algorithm with 200,000 evaluations of *D* dimensional Elliptic Function. *T*_2_ is evaluated five times, and ${\hat{T}}_{2}$ is used to denote the mean *T*_2_. At last, the complexity of the algorithm is reflected by: *T*_0_, *T*_1_, ${\hat{T}}_{2}$, ${(\hat{T}}_{2}-T_{1})/T_{0}$.

### 4.3 Sensitivity analysis to control parameters

#### 4.3.1 Sensitivity analysis to population size

**Fig 2:**
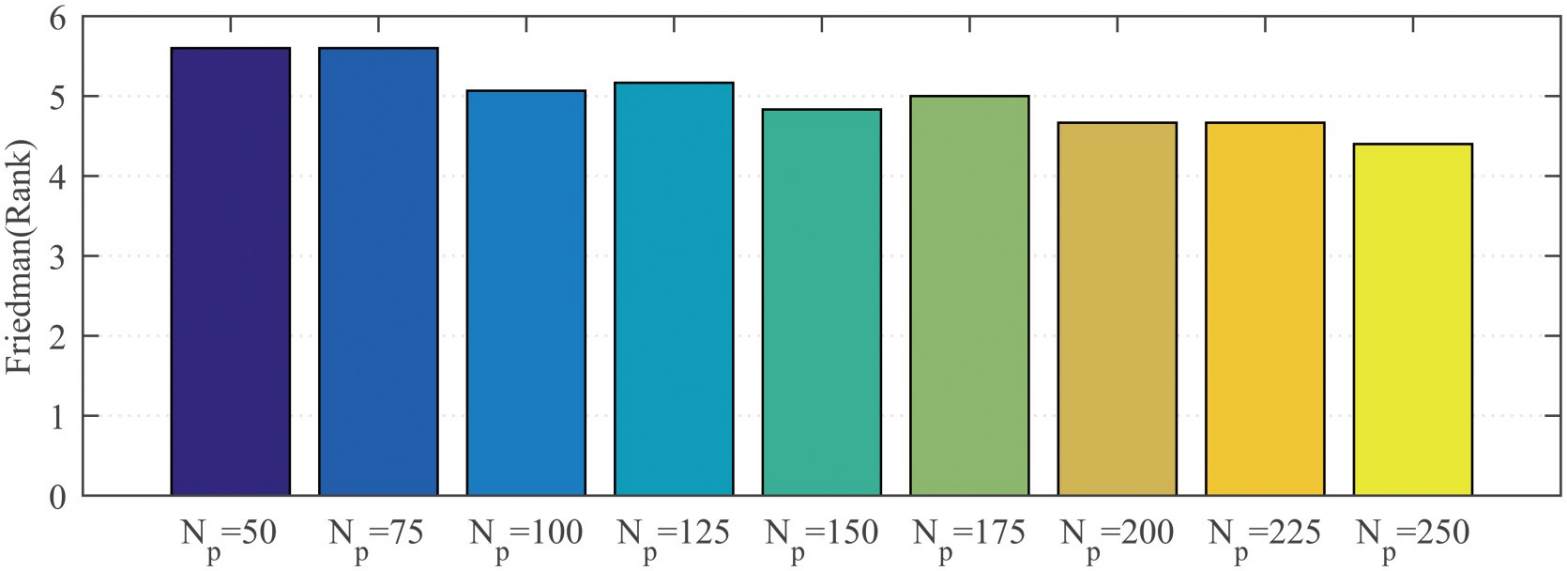
Friedman test results at *D* = 30 over 30 independent runs.

The effect of population size in SaDSDE is investigated by testing 30-dimensional problems. *N*_*p*_ is set to 50, 75, 100, 125, 150, 175, 200, 225 and 250. For each test function with a different population size, the maximum generation number is still set to 1000. From the statistical Friedman test [50] results in Fig 2, the performance of SaDSDE decreases with reducing population size, because a larger population size has high exploration ability.

Although SaDSDE with *N*_*p*_ = 250 is best in Fig 2, there is no obvious performance difference in Table 3. Therefore, we can say that SaDSDE is not sensitive to the population size.

**Table 3:** The results of Wilcoxon’s rank-sum test over 30 independent runs.

Comparisons	R+	R-	*p*-value	*α* = 0.05	*α* = 0.1
**SaDSDE with *N***_***p***_ **= 250 versus SaDSDE with *N***_***p***_ **= 50**	21	0	9.17E-01	No	No
**SaDSDE with *N***_***p***_ **= 250 versus SaDSDE with *N***_***p***_ **= 75**	16	5	9.32E-01	No	No
**SaDSDE with *N***_***p***_ **= 250 versus SaDSDE with *N***_***p***_ **= 100**	21	0	9.62E-01	No	No
**SaDSDE with *N***_***p***_ **= 250 versus SaDSDE with *N***_***p***_ **= 125**	15	6	9.62E-01	No	No
**SaDSDE with *N***_***p***_ **= 250 versus SaDSDE with *N***_***p***_ **= 150**	18	3	9.77E-01	No	No
**SaDSDE with *N***_***p***_ **= 250 versus SaDSDE with *N***_***p***_ **= 175**	15	6	9.77E-01	No	No
**SaDSDE with *N***_***p***_ **= 250 versus SaDSDE with *N***_***p***_ **= 200**	21	0	9.62E-01	No	No
**SaDSDE with *N***_***p***_ **= 250 versus SaDSDE with *N***_***p***_ **= 225**	10	11	9.92E-01	No	No

Sign “No” indicates that the performance of SaDSDE with *N*_*p*_ = 250 is similar to its competitor.

#### 4.3.2 Sensitivity analysis to crossover probability

**Fig 3:**
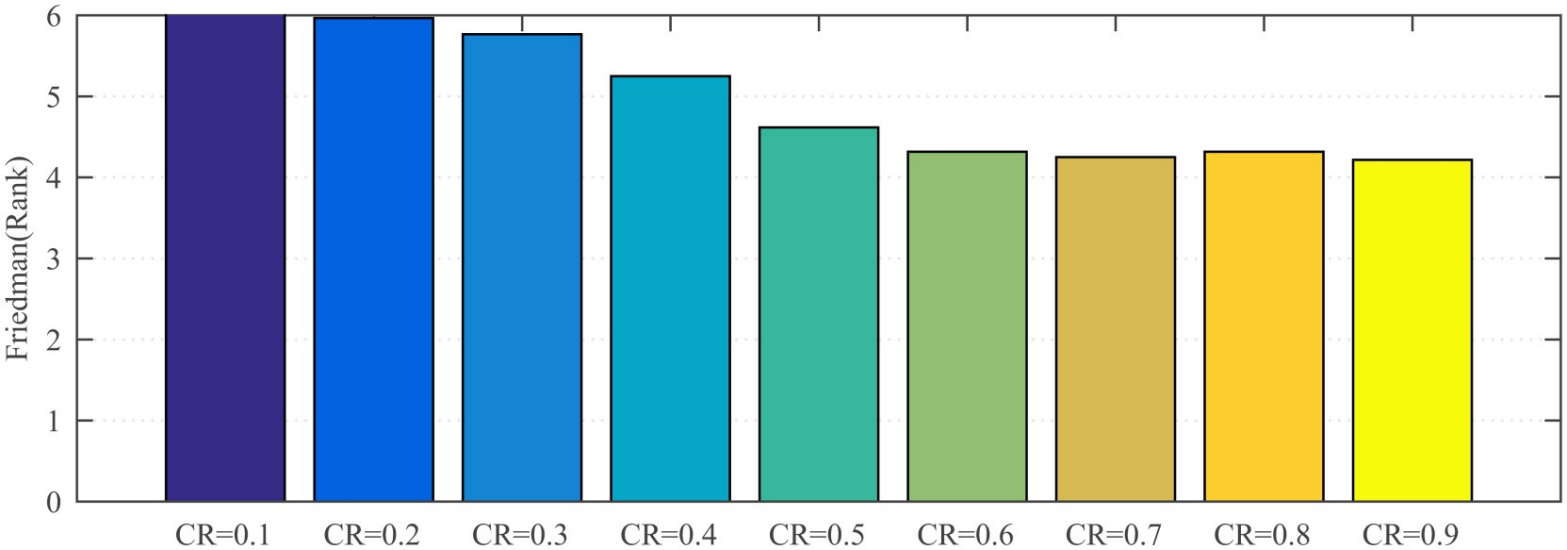
The result of Friedman test with 30 variables over 30 independent runs.

The impact of the crossover probability *CR* on the performance of proposed algorithm is also analyzed. The candidate values of *CR* are 0.1, 0.2, 0.3, 0.4, 0.5, 0.6, 0.7, 0.8 and 0.9, respectively. We perform Friedman test and Wilcoxon’s rank-sum test [50] among the optimization result on different *CR* values, respectively. The test results are shown in Fig 3 and Table 4, respectively. From Fig 3, we can observe that the performance of SaDSDE is best at *CR* = 0.9.

**Table 4:** The results of Wilcoxon’s rank-sum test over 30 independent runs.

Comparisons	R+	R-	*p*-value	*α* = 0.05	*α* = 0.1
**SaDSDE with *CR* = 0.9 versus SaDSDE with *CR* = 0.1**	110	80	6.68E-02	No	Yes
**SaDSDE with *CR* = 0.9 versus SaDSDE with *CR* = 0.2**	109	81	6.68E-02	No	Yes
**SaDSDE with *CR* = 0.9 versus SaDSDE with *CR* = 0.3**	123	67	7.94E-02	No	Yes
**SaDSDE with *CR* = 0.9 versus SaDSDE with *CR* = 0.4**	108	63	1.11E-01	No	No
**SaDSDE with *CR* = 0.9 versus SaDSDE with *CR* = 0.5**	30	36	5.83E-01	No	No
**SaDSDE with *CR* = 0.9 versus SaDSDE with *CR* = 0.6**	21	45	6.06E-01	No	No
**SaDSDE with *CR* = 0.9 versus SaDSDE with *CR* = 0.7**	22	33	7.39E-01	No	No
**SaDSDE with *CR* = 0.9 versus SaDSDE with *CR* = 0.8**	29	7	7.22E-01	No	No

R+ is the sum of ranks in which the first algorithm outperformed the second, and R- is the sum of ranks for the opposite. The R+ is bigger, the first algorithm is better. “Yes” indicates that the performance of the SaDSDE with *CR* = 0.9 is better than its competitor significantly, “No” indicates that there is no significant performance discrepancy.

From Table 4, it can be observed that SaDSDE is not sensitive to *CR* except for *CR* = {0.1, 0.2, 0.3} at the 0.1 significance level. Based on the trade-off of the convergence precision and convergence rate, we think that *CR* = 0.9 is a more appropriate value. Therefore, we set *CR* = 0.9 in the following series of experiments unless noted otherwise.

### 4.4 Parameter settings and involved algorithms

For rigorous performance verification, SaDSDE is compared with the following 7 well-established DE variants (i.e., Rcr-JADE [11], EsDEr-NR [17], IMMSADE [18], AGDE [21], EFADE [23], MPEDE [29] and EDEV [32]) and 3 non-DE algorithms (i.e., social learning particle swarm optimization (SL-PSO) [51], grey wolf optimization (GWO) [52] and whale optimization algorithm (WOA) [53]). Most parameters of compared algorithms are kept the same as used in their original literatures, which are shown in Table 5.

**Table 5:** Parameter settings.

Algorithm	Parameters
**Rcr-JADE**	*μ*_*F*_ = *μ*_*CR*_ = 0.5, *c* = 0.1, *p* = 0.5, *F* = randn(*μ*_*F*_, 0.1), *CR* = randn(*μ*_*CR*_, 0.1)
**EsDEr-NR**	*μ*_*F*_ = *μ*_*CR*_ = *freq* = 0.5, *G*_*LS*_ = 250, *Np*_*max*_ = 18 * D, *Np*_*min*_ = 4
**IMMSADE**	*Τ* = 0.7, *λ*ϵ[0.7, 1.0], *F*ϵ[0.1, 0.8], *CR*ϵ[0.3, 1.0]
**AGDE**	*CR*_1_ ϵ[0.05, 0.15], *CR*_2_ ϵ[0.9, 1.0], *F* ϵ[0.1, 1.0], *p* = 0.1
**EFADE**	*ε* = 0.01, *CR*_1_ ϵ[0.05, 0.15], *CR*_2_ ϵ[0.9, 1.0]
**MPEDE**	*c* = 0.1, *p* = 0.4, *λ*_1_ = *λ*_2_ = *λ*_3_
**EDEV**	*λ*_1_ = *λ*_2_ = *λ*_3_ = 0.1, *λ*_4_ = 0.7, *ng* = 20
**SL-PSO**	*α* = 0.5, *β* = 0.01
**GWO**	$a=2-\left(\frac{2}{T}\right),\;A=2*a*\text{rand}()-a,C=2*\text{rand}()$
**WOA**	*$p=\text{rand}(),a=2-t*\left(\frac{2}{T}\right),\;A=2*a*\text{rand}()-a,C=2*\text{rand}()$*

The common parameters are set as follows. The max number of iterations is set to *T* = 1000, the population size is set to *N*_*p*_ = 100, and the times of all experiment runs is set to 30.

### 4.5 Experimental comparisons

#### 4.5.1 Numerical analysis

The optimization performance of DE is evaluated in terms of the Mean and Standard Deviation (STD) of the function solutions, and the best and the second best results are respectively marked in bold and italic. In order to show the performance clearly, the number of the best and second best results are summarize graphically. In addition, 4 non-parametric statistical tests [50] are used to compare the performances among all the algorithms, including Wilcoxon signed-rank test, Friedman test, Kruskal_Wallis test and Wilcoxon rank-sum test. Friedman test and Kruskal_Wallis test are conducted based on the optimization results to evaluate the overall performance and we can obtain the rankings of algorithms. Wilcoxon signed-rank test at the 0.05 significance level and Wilcoxon rank-sum test respectively are used to verify the differences among algorithms for single and multiple problems. Signs “+”, “-” and “≈” indicate that the performance of SaDSDE is significantly better than, worse than and similar to its competitor, respectively. R^+^ denotes the sum of ranks for the test problems in which the first algorithm performs better than the second algorithm, and R^−^ represents the sum of ranks for the test problems in which the first algorithm performs worse than the second algorithm. Larger ranks indicate larger performance discrepancy. “*p*-value” indicates the probability of rejecting the hypothesis. “Yes” indicates that the performance of the first algorithm is better than the second algorithm significantly, and “No” indicates that there is no significant performance discrepancy.

#### A. Comparison with 7 improved DE variants

**Fig 4:**
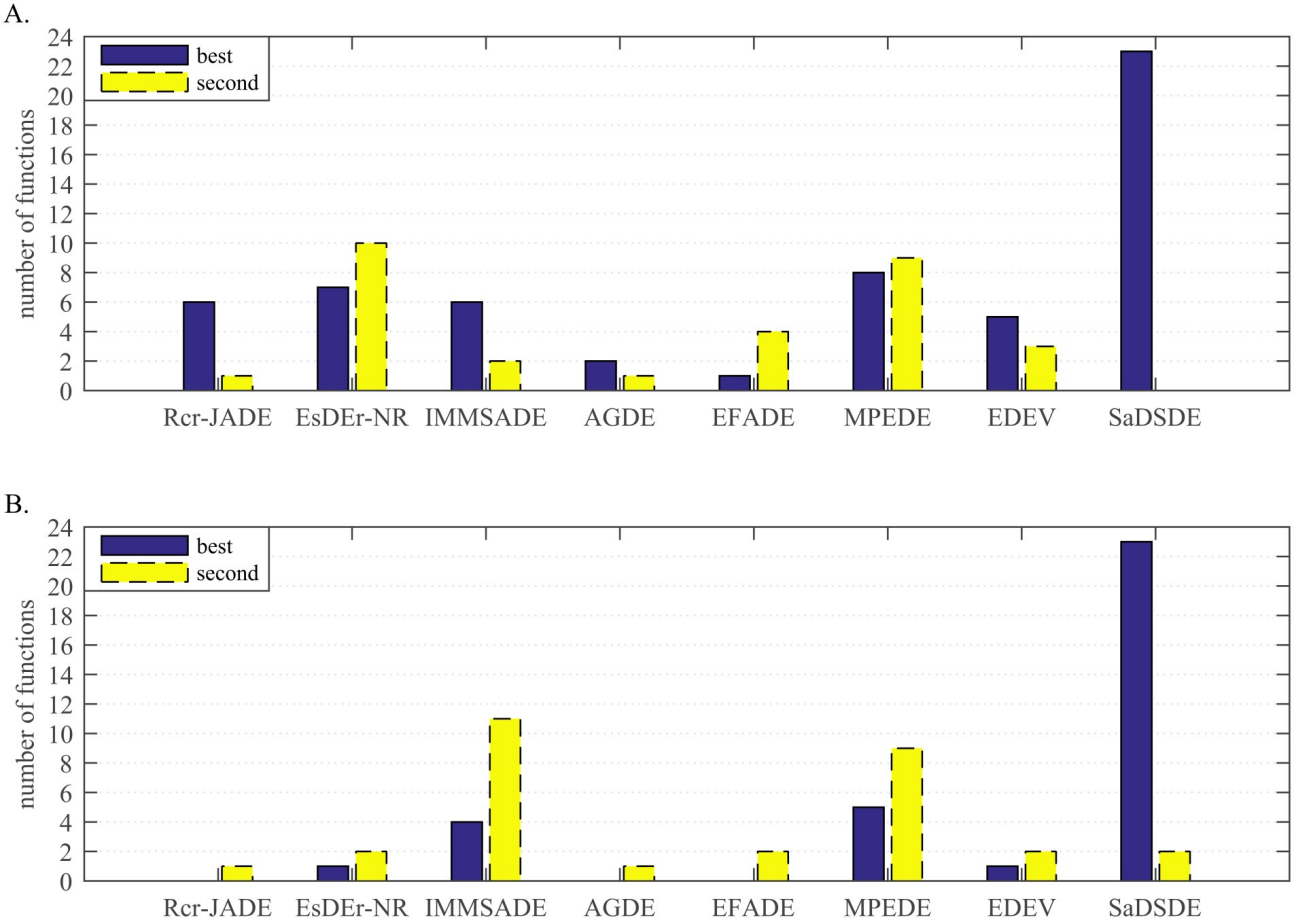
Number of cases on which each algorithm performs the best and second best in the comparison. (A) *D* = 30. (B) *D* = 100.

**Fig 5:**
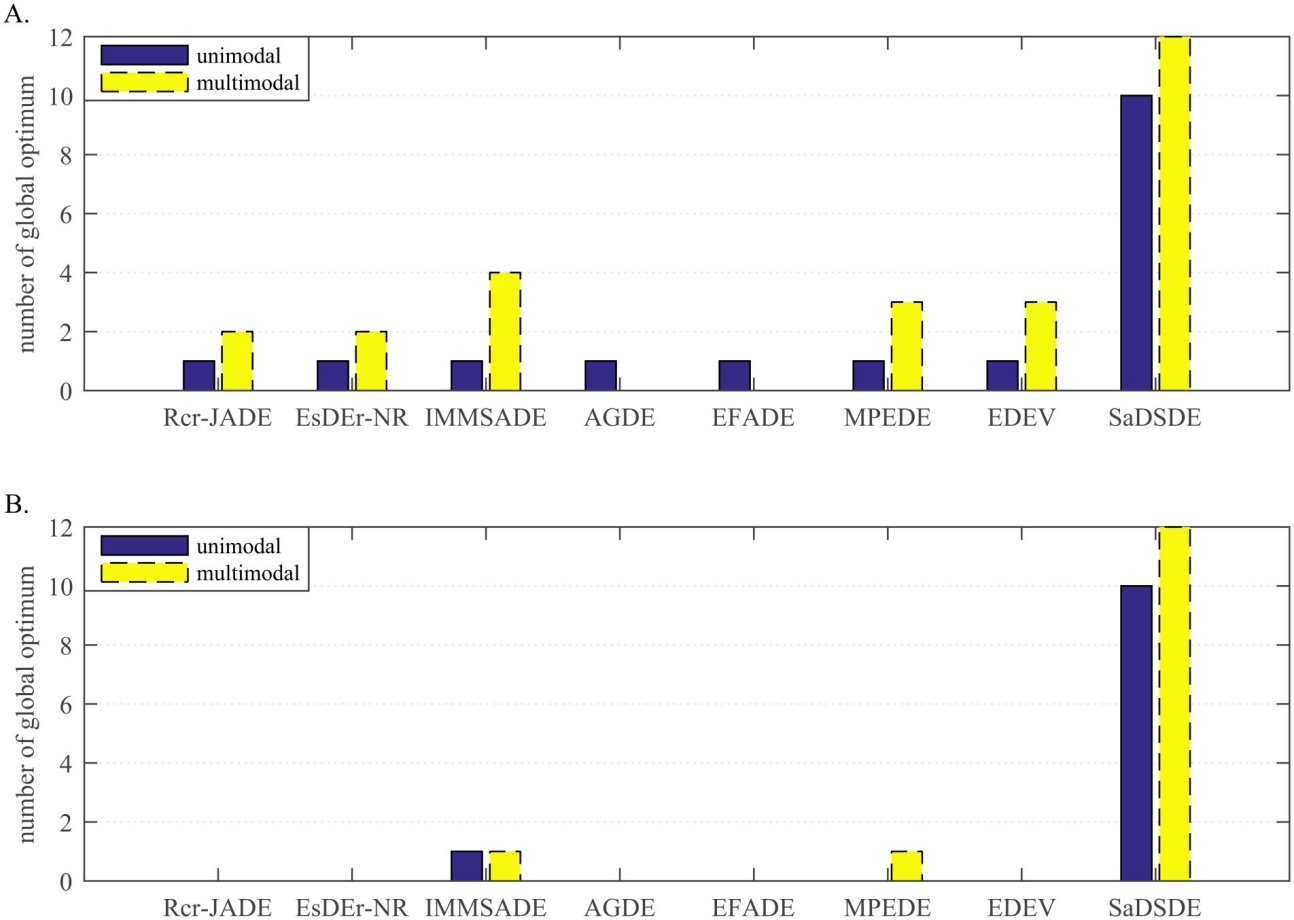
Number of cases on which each algorithm obtains global optimum. (A) *D* = 30. (B) *D* = 100.

**Fig 6:**
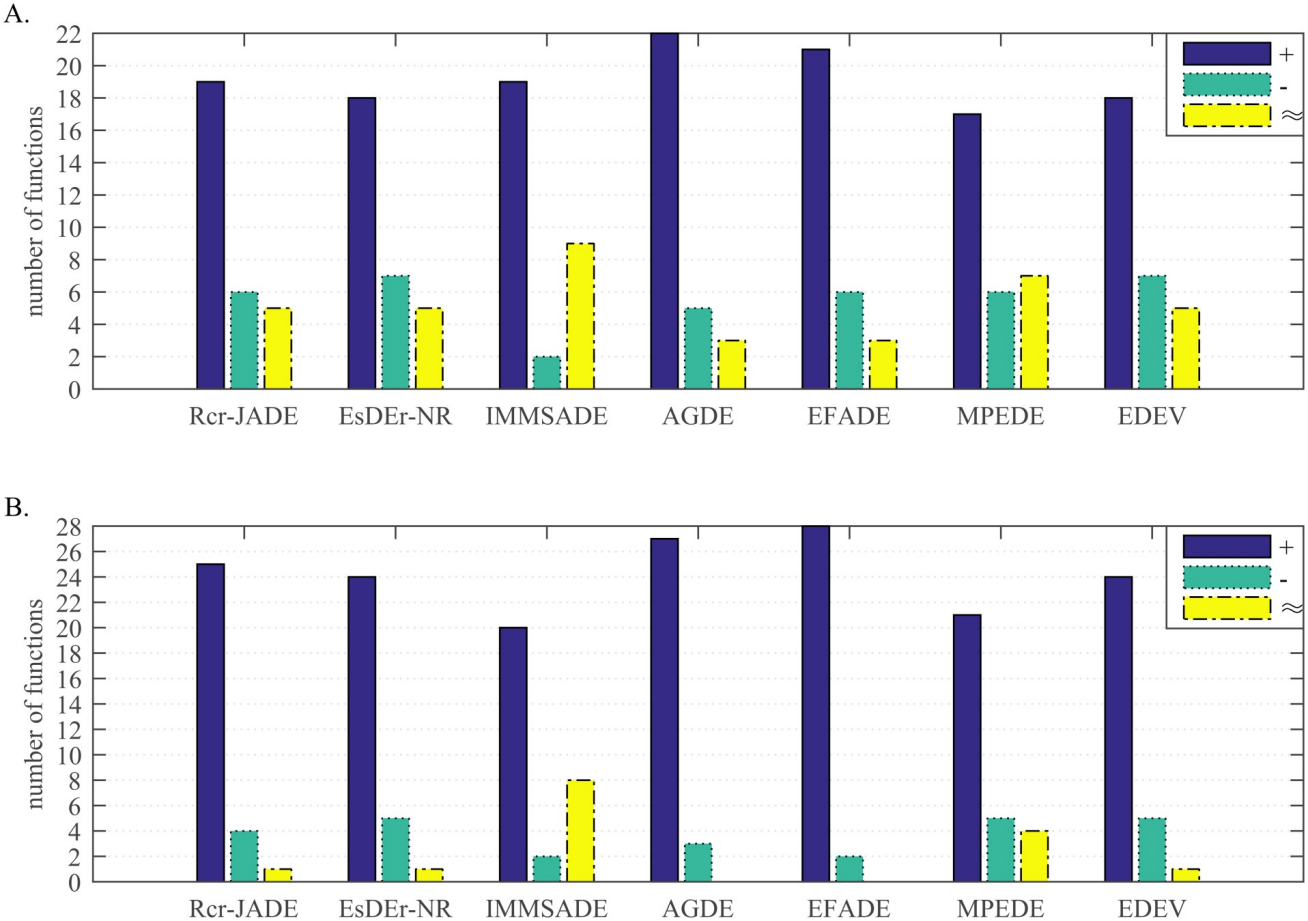
Wilcoxon signed-rank test results at the 0.05 significance level. (A) *D* = 30. (B) *D* = 100.

**Fig 7:**
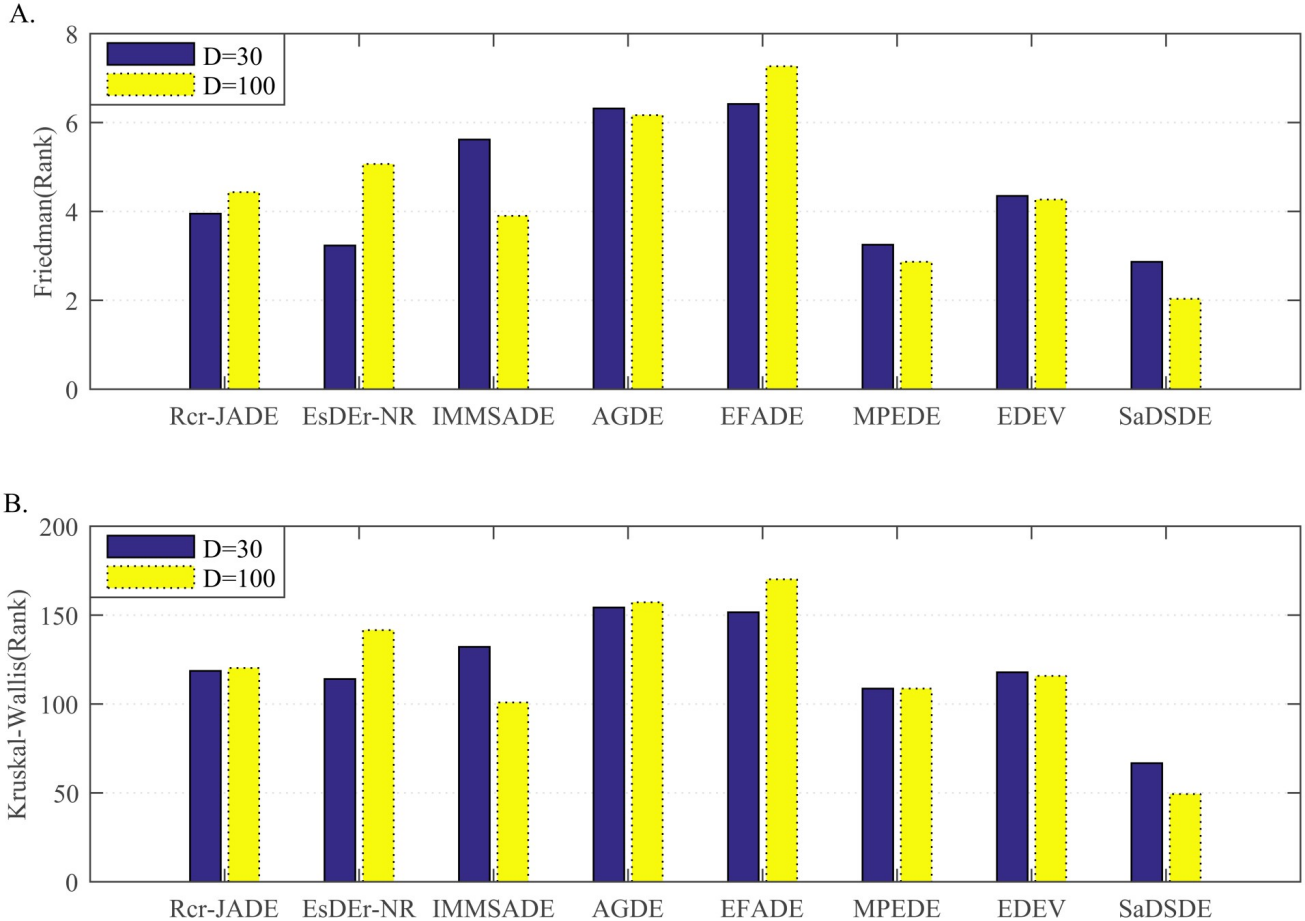
Non-parametric statistical test results over 30 independent runs. (A) Friedman test results. (B) Kruskal_Wallis test results.

In this experiment, the proposed algorithm is compared with 7 improved DE variants on 30-dimensional and 100-dimensional problems. The experimental results are listed in Tables 6 and 7. To show the performance clearly, we summarize the number of the best, the second best results and global optimums graphically in Figs 4 and 5, respectively. Wilcoxon signed-rank test results and the average rankings of all the DEs are shown in Figs 6 and 7, respectively. In addition, Wilcoxon rank-sum test results are represented in Table 8.

**Table 6:** The optimization results obtained by SaDSDE and 7 DE variants at *D* = 30.

*F*	Rcr-JADE	EsDEr-NR	IMMSADE	AGDE	EFADE	MPEDE	EDEV	SaDSDE
	Mean ± STD	Mean ± STD	Mean ± STD	Mean ± STD	Mean ± STD	Mean ± STD	Mean ± STD	Mean ± STD
***f***_***1***_	3.52E-51±1.06E-50	*1*.*80E-54±1*.*22E-54*	4.41E-28±1.67E-27	2.25E-09±6.18E-10	3.64E-12±5.56E-12	7.76E-36±2.31E-35	2.54E-31±1.09E-30	**0.00E+00±0.00E+00**
***f***_***2***_	1.74E-13±4.67E-13	1.32E-11±3.14E-11	6.51E+00±1.63E+01	1.72E+00±9.76E-01	2.52E+00±2.63E+00	*1*.*70E-21±8*.*93E-21*	1.19E-07±3.35E-07	**0.00E+00±0.00E+00**
***f***_***3***_	3.75E-46±9.37E-46	*3*.*04E-50±4*.*19E-50*	8.56E-26±4.67E-25	5.24E-06±2.57E-06	4.69E-10±4.27E-10	1.55E-31±4.74E-31	1.40E-26±7.39E-26	**0.00E+00±0.00E+00**
***f***_***4***_	1.12E-24±2.60E-24	*4*.*60E-26±2*.*88E-26*	7.96E-17±3.30E-16	2.42E-06±3.51E-07	2.80E-05±6.21E-06	2.19E-17±3.78E-17	1.18E-14±4.36E-14	**0.00E+00±0.00E+00**
***f***_***5***_	2.09E-11±2.50E-11	5.33E-13±1.44E-12	2.72E-08±4.78E-08	1.18E+00±1.74E-01	2.72E+00±4.92E-01	*5*.*39E-14±4*.*81E-14*	1.94E-02±1.82E-02	**0.00E+00±0.00E+00**
***f***_***6***_	9.28E-52±1.68E-51	*3*.*07E-55±2*.*46E-55*	4.15E-31±1.46E-30	2.53E-10±9.49E-11	2.08E-13±2.06E-13	1.28E-37±5.09E-37	5.82E-31±2.72E-30	**0.00E+00±0.00E+00**
***f***_***7***_	8.73E-45±2.61E-44	*2*.*69E-48±2*.*55E-48*	9.11E-25±3.50E-24	2.39E-03±6.11E-04	3.65E-06±8.10E-06	1.93E-31±3.95E-31	3.58E-26±1.63E-25	**0.00E+00±0.00E+00**
***f***_***8***_	3.44E-10±7.06E-10	4.36E-09±1.12E-08	4.02E+00±3.42E+00	3.41E-09±7.38E-10	*9*.*11E-13±1*.*11E-12*	1.42E-06±6.54E-06	2.42E-05±8.19E-05	**0.00E+00±0.00E+00**
***f***_***9***_	3.81E-44±8.30E-44	*5*.*54E-47±1*.*10E-46*	1.02E-20±5.50E-20	1.52E-03±4.89E-04	1.78E-06±3.36E-06	3.43E-30±9.72E-30	1.26E-26±2.58E-26	**0.00E+00±0.00E+00**
***f***_***10***_	**0.00E+00±0.00E+00**	**0.00E+00±0.00E+00**	**0.00E+00±0.00E+00**	**0.00E+00±0.00E+00**	**0.00E+00±0.00E+00**	**0.00E+00±0.00E+00**	**0.00E+00±0.00E+00**	**0.00E+00±0.00E+00**
***f***_***11***_	1.25E+03±8.23E-06	**2.13E-03±7.04E-04**	1.25E+03±5.75E-06	2.01E-02±4.87E-03	4.50E-03±1.77E-03	1.25E+03±5.04E-06	*2*.*56E-03±7*.*91E-04*	1.25E+03±3.83E-06
***f***_***12***_	3.15E-02±2.32E-02	6.16E-02±2.25E-01	4.13E+01±3.25E+01	7.32E+00±2.83E+00	2.60E+01±4.49E+00	*3*.*13E-11±3*.*41E-11*	6.07E-05±2.45E-04	**0.00E+00±0.00E+00**
***f***_***13***_	**0.00E+00±0.00E+00**	**0.00E+00±0.00E+00**	**0.00E+00±0.00E+00**	1.07E-06±3.25E-06	*1*.*37E-09±1*.*18E-09*	2.47E-04±1.35E-03	**0.00E+00±0.00E+00**	**0.00E+00±0.00E+00**
***f***_***14***_	**0.00E+00±0.00E+00**	**0.00E+00±0.00E+00**	**0.00E+00±0.00E+00**	9.19E-14±2.57E-14	*1*.*07E-16±5*.*88E-16*	**0.00E+00±0.00E+00**	**0.00E+00±0.00E+00**	**0.00E+00±0.00E+00**
***f***_***15***_	3.30E-52±5.98E-52	*2*.*50E-55±2*.*54E-55*	1.05E-26±5.75E-26	6.44E-10±2.30E-10	2.53E-12±2.00E-12	3.55E-38±6.14E-38	6.53E-30±3.55E-29	**0.00E+00±0.00E+00**
***f***_***16***_	5.68E-15±1.77E-15	*3*.*67E-15±6*.*49E-16*	3.79E-15±1.30E-15	1.13E-05±1.48E-06	8.46E-05±2.09E-05	*3*.*67E-15±6*.*49E-16*	3.79E-15±9.01E-16	**0.00E+00±0.00E+00**
***f***_***17***_	**3.15E-01±1.02E+00**	1.11E+01±1.95E+00	2.59E+01±1.53E-01	4.13E+01±2.94E+01	3.17E+01±2.10E+01	*5*.*63E-01±1*.*38E+00*	5.71E+00±1.58E+00	2.35E+01±6.98E-01
***f***_***18***_	8.28E-02±5.98E-02	9.30E-02±1.66E-01	*2*.*42E-05±5*.*63E-05*	9.75E-01±1.16E-01	4.52E+00±3.29E-01	3.64E-03±6.38E-03	1.71E-01±8.76E-02	**0.00E+00±0.00E+00**
***f***_***19***_	**3.82E-04±0.00E+00**	**3.82E-04±0.00E+00**	**3.82E-04±0.00E+00**	**3.82E-04±5.40E-11**	**3.82E-04±2.88E-09**	**3.82E-04±0.00E+00**	**3.82E-04±0.00E+00**	**3.82E-04±0.00E+00**
***f***_***20***_	*2*.*22E-01±3*.*61E-02*	2.36E-01±3.32E-02	4.34E-01±6.80E-02	4.24E-01±3.81E-02	4.74E-01±6.65E-02	**2.18E-01±2.58E-02**	2.67E-01±3.78E-02	5.29E-01±8.87E-02
***f***_***21***_	3.83E-01±1.18E-01	3.73E-01±1.36E-01	3.97E-01±3.27E-02	**2.79E-01±2.39E-02**	3.32E-01±2.92E-02	*3*.*20E-01±8*.*86E-02*	3.59E-01±1.08E-01	5.00E-01±0.00E+00
***f***_***22***_	5.59E-04±1.70E-03	1.39E-03±5.45E-03	**0.00E+00±0.00E+00**	8.61E-04±1.29E-04	7.15E-03±7.52E-04	**0.00E+00±0.00E+00**	*1*.*01E-07±4*.*34E-07*	**0.00E+00±0.00E+00**
***f***_***23***_	5.15E-03±7.43E-04	5.01E-03±1.21E-03	6.01E-01±8.73E-02	1.14E-01±1.17E-02	1.14E-01±1.50E-02	*1*.*34E-03±3*.*70E-04*	1.00E-02±1.74E-03	**0.00E+00±0.00E+00**
***f***_***24***_	4.09E+00±3.25E-01	*4*.*85E-01±6*.*53E-02*	8.70E+00±4.92E-01	1.23E+00±9.76E-02	4.80E+00±3.90E-01	2.03E+00±3.35E-01	1.22E+00±1.98E-01	**0.00E+00±0.00E+00**
***f***_***25***_	4.14E+00±3.30E-01	**2.08E+00±1.82E-01**	1.19E+01±1.08E+00	5.71E+00±3.97E-01	6.71E+00±7.35E-01	*2*.*32E+00±2*.*58E-01*	3.54E+00±5.65E-01	1.25E+01±7.66E-01
***f***_***26***_	8.46E+00±1.30E+00	6.31E-01±5.86E-01	5.20E+01±7.55E+00	1.57E+01±8.42E-01	2.11E+01±1.13E+00	*4*.*81E-04±7*.*07E-04*	5.95E-01±5.12E-01	**0.00E+00±0.00E+00**
***f***_***27***_	1.02E-03±7.39E-04	6.29E-07±3.44E-06	*4*.*20E-15±1*.*23E-14*	3.77E-03±4.32E-04	9.22E-03±8.62E-04	1.58E-05±1.52E-05	1.82E-03±6.17E-04	**0.00E+00±0.00E+00**
***f***_***28***_	3.50E-02±1.92E-01	1.38E-02±7.54E-02	**0.00E+00±0.00E+00**	2.17E-07±7.59E-08	*1*.*41E-08±1*.*73E-08*	**0.00E+00±0.00E+00**	**0.00E+00±0.00E+00**	**0.00E+00±0.00E+00**
***f***_***29***_	**1.57E-32±5.57E-48**	**1.57E-32±5.57E-48**	1.68E-03±6.77E-04	2.95E-11±1.40E-11	1.51E-12±2.76E-12	**1.57E-32±5.57E-48**	*1*.*72E-31±4*.*26E-31*	1.34E-10±2.75E-10
***f***_***30***_	1.68E-32±1.40E-33	*1*.*37E-32±4*.*26E-34*	4.72E-03±1.89E-03	6.35E-13±2.51E-13	4.89E-16±6.39E-16	**1.35E-32±5.57E-48**	1.34E-31±4.59E-31	1.24E-01±3.61E-01

**Table 7:** The optimization results obtained by SaDSDE and 7 DE variants at *D* = 100.

*F*	Rcr-JADE	EsDEr-NR	IMMSADE	AGDE	EFADE	MPEDE	EDEV	SaDSDE
	Mean ± STD	Mean ± STD	Mean ± STD	Mean ± STD	Mean ± STD	Mean ± STD	Mean ± STD	Mean ± STD
***f***_***1***_	1.36E-15±1.51E-15	5.47E-04±1.44E-03	*4*.*76E-17±1*.*81E-16*	3.97E+00±7.32E-01	2.56E+01±1.33E+01	1.74E-16±3.07E-16	8.17E-10±6.88E-10	**0.00E+00±0.00E+00**
***f***_***2***_	1.99E+02±6.49E+01	6.55E+02±1.97E+02	1.22E+04±1.44E+04	5.84E+04±3.33E+04	9.93E+03±2.34E+03	*7*.*22E+00±3*.*38E+00*	3.92E+02±1.47E+02	**0.00E+00±0.00E+00**
***f***_***3***_	8.36E-10±1.13E-09	1.12E+03±2.52E+03	*5*.*96E-12±2*.*85E-11*	5.77E+03±9.39E+02	4.38E+03±2.61E+03	7.56E-11±9.07E-11	2.23E-06±1.63E-06	**0.00E+00±0.00E+00**
***f***_***4***_	2.58E-06±4.13E-06	7.09E-02±7.71E-02	*1*.*73E-09±4*.*51E-09*	7.55E-01±5.60E-02	2.59E+00±4.60E-01	4.42E-08±3.95E-08	2.05E-06±3.04E-06	**0.00E+00±0.00E+00**
***f***_***5***_	8.01E+00±1.32E+00	1.01E+01±1.92E+00	*5*.*71E-04±1*.*39E-03*	4.04E+01±1.73E+00	5.03E+01±3.01E+00	3.97E-01±1.20E-01	1.20E+01±1.60E+00	**0.00E+00±0.00E+00**
***f***_***6***_	1.16E-15±2.05E-15	4.77E-04±1.15E-03	3.44E-13±1.88E-12	1.52E+00±2.11E-01	9.31E+00±5.25E+00	*5*.*73E-17±8*.*31E-17*	2.82E-10±1.74E-10	**0.00E+00±0.00E+00**
***f***_***7***_	3.98E-09±4.50E-09	7.89E+02±2.54E+03	*2*.*08E-11±1*.*04E-10*	3.88E+06±7.56E+05	2.57E+07±1.11E+07	1.35E-10±1.39E-10	1.79E-04±1.71E-04	**0.00E+00±0.00E+00**
***f***_***8***_	4.88E+00±2.34E+00	5.02E+00±1.14E+01	8.63E+01±4.07E+01	4.65E-01±1.94E-01	*2*.*23E-01±1*.*35E-01*	1.19E+01±1.71E+01	1.22E+00±1.55E+00	**0.00E+00±0.00E+00**
***f***_***9***_	2.49E-08±3.31E-08	9.87E+02±1.28E+03	*5*.*01E-10±2*.*54E-09*	3.77E+06±7.22E+05	2.29E+07±1.33E+07	5.77E-09±1.59E-08	2.48E-04±3.05E-04	**0.00E+00±0.00E+00**
***f***_***10***_	6.47E+00±4.86E+00	7.61E+01±2.96E+01	**0.00E+00±0.00E+00**	2.70E+00±1.82E+00	3.20E+01±1.21E+01	*1*.*97E+00±1*.*71E+00*	3.20E+00±5.62E+00	**0.00E+00±0.00E+00**
***f***_***11***_	1.36E+04±6.12E-06	**5.25E-02±1.30E-02**	1.36E+04±5.84E-06	2.43E-01±4.77E-02	*6*.*48E-02±1*.*57E-02*	1.36E+04±4.05E-06	8.02E-02±1.49E-02	1.36E+04±5.13E-06
***f***_***12***_	2.02E+02±1.12E+01	1.02E+02±9.02E+00	1.36E+02±2.20E+02	4.52E+02±1.36E+01	5.61E+02±1.45E+01	*4*.*48E+01±5*.*65E+00*	1.97E+02±1.60E+01	**0.00E+00±0.00E+00**
***f***_***13***_	3.28E-03±5.50E-03	1.73E-02±3.31E-02	*3*.*38E-15±1*.*60E-14*	9.53E-01±5.43E-02	1.38E+00±1.36E-01	4.02E-03±7.30E-03	7.11E-03±1.52E-02	**0.00E+00±0.00E+00**
***f***_***14***_	*2*.*35E-16±1*.*91E-16*	3.37E-08±1.36E-07	**0.00E+00±0.00E+00**	2.17E-04±3.38E-05	1.25E-03±8.02E-04	**0.00E+00±0.00E+00**	5.87E-14±2.84E-14	**0.00E+00±0.00E+00**
***f***_***15***_	1.19E-15±1.61E-15	7.59E-05±1.19E-04	1.46E-15±7.94E-15	1.08E+00±1.11E-01	4.52E+00±1.19E+00	*2*.*27E-17±4*.*40E-17*	1.90E-10±7.61E-11	**0.00E+00±0.00E+00**
***f***_***16***_	1.29E+00±3.98E-01	2.75E+00±3.43E-01	*9*.*97E-10±2*.*30E-09*	4.26E-01±4.69E-02	3.91E+00±2.44E-01	1.61E-01±3.68E-01	1.37E+00±4.04E-01	**0.00E+00±0.00E+00**
***f***_***17***_	1.42E+02±4.35E+01	3.42E+02±1.50E+02	**9.69E+01±2.86E-01**	2.46E+04±5.24E+03	1.08E+03±4.62E+02	1.13E+02±3.47E+01	3.33E+02±1.50E+02	*9*.*70E+01±6*.*77E-01*
***f***_***18***_	2.75E+01±1.38E+01	1.01E+02±1.52E+01	*8*.*48E-03±1*.*44E-02*	1.65E+02±6.67E+00	3.72E+02±1.57E+01	2.97E+01±9.40E+00	1.25E+01±4.45E+00	**0.00E+00±0.00E+00**
***f***_***19***_	**1.27E-03±5.03E-12**	*1*.*30E-03±6*.*94E-05*	**1.27E-03±0.00E+00**	4.27E-01±6.59E-02	3.19E+00±1.55E+00	**1.27E-03±1.33E-12**	**1.27E-03±3.26E-11**	**1.27E-03±0.00E+00**
***f***_***20***_	5.31E-01±6.62E-02	6.57E-01±6.67E-02	8.54E-01±6.00E-02	7.96E-01±6.00E-02	2.31E+00±9.65E-01	**4.94E-01±5.71E-02**	*5*.*22E-01±6*.*80E-02*	9.31E-01±6.87E-02
***f***_***21***_	6.04E-01±2.01E-01	5.44E-01±2.46E-01	5.01E-01±6.34E-02	7.11E-01±2.89E-01	2.12E+01±1.42E+01	**4.97E-01±1.63E-01**	5.57E-01±1.93E-01	*5*.*00E-01±0*.*00E+00*
***f***_***22***_	4.54E+00±1.79E+00	1.29E+01±2.24E+00	*1*.*54E-06±6*.*70E-06*	4.19E+00±2.68E-01	1.99E+01±1.48E+00	3.43E+00±1.10E+00	1.71E+00±8.96E-01	**0.00E+00±0.00E+00**
***f***_***23***_	1.17E-01±1.16E-02	1.08E-01±1.53E-02	2.00E+00±1.58E-01	5.71E-01±4.70E-02	5.87E-01±4.06E-02	*1*.*35E-02±2*.*97E-03*	1.87E-01±5.13E-02	**0.00E+00±0.00E+00**
***f***_***24***_	3.35E+01±5.89E-01	*5*.*78E+00±4*.*78E-01*	4.20E+01±7.16E-01	1.83E+01±6.23E-01	3.81E+01±4.67E-01	2.46E+01±1.70E+00	1.50E+01±4.21E+00	**0.00E+00±0.00E+00**
***f***_***25***_	3.67E+01±2.09E+00	*2*.*15E+01±1*.*62E+00*	6.67E+01±7.36E+00	5.84E+01±1.76E+00	9.89E+01±2.59E+01	**2.13E+01±2.16E+00**	3.91E+01±2.64E+00	4.39E+01±7.11E-01
***f***_***26***_	1.47E+02±6.66E+00	7.72E+01±4.76E+00	4.75E+02±9.81E+01	3.49E+02±2.30E+01	4.22E+02±2.11E+01	*6*.*95E+01±6*.*31E+00*	1.40E+02±8.54E+00	**0.00E+00±0.00E+00**
***f***_***27***_	1.99E-06±9.67E-06	7.80E-03±9.03E-03	1.43E-08±7.61E-08	2.50E+00±6.58E-01	4.82E+01±2.03E+00	*1*.*08E-08±1*.*56E-08*	7.27E-04±1.74E-03	**0.00E+00±0.00E+00**
***f***_***28***_	8.89E+00±2.09E+00	2.21E+01±3.01E+00	*4*.*68E-15±2*.*50E-14*	4.16E+01±2.13E+00	1.21E+02±4.60E+01	5.22E+00±1.68E+00	7.73E+00±2.00E+00	**0.00E+00±0.00E+00**
***f***_***29***_	1.56E-03±8.57E-03	7.21E-05±2.34E-04	3.03E-02±9.81E-03	1.22E-01±2.64E-02	4.31E-01±1.38E-01	**8.74E-20±1.10E-19**	*3*.*30E-11±1*.*23E-10*	3.96E-02±2.27E-02
***f***_***30***_	2.56E-03±4.73E-03	3.66E-03±5.27E-03	3.45E-01±9.96E-02	*1*.*92E-03±2*.*83E-04*	8.57E-03±5.20E-03	2.56E-03±4.73E-03	**1.39E-03±5.91E-03**	9.33E+00±1.73E+00

**Table 8:** The results of Wilcoxon rank-sum test over 30 independent runs.

Comparisons	*D* = 30					*D* = 100				
	R+	R-	*p*-value	*α* = 0.05	*α* = 0.1	R+	R-	*p*-value	*α* = 0.05	*α* = 0.1
**SaDSDE versus Rcr-JADE**	227	124	6.19E-04	Yes	Yes	391	74	6.94E-06	Yes	Yes
**SaDSDE versus EsDEr-NR**	205	146	9.15E-04	Yes	Yes	379	86	1.37E-06	Yes	Yes
**SaDSDE versus IMMSADE**	238	62	5.76E-04	Yes	Yes	303	75	1.04E-04	Yes	Yes
**SaDSDE versus AGDE**	323	112	3.84E-05	Yes	Yes	419	46	2.24E-07	Yes	Yes
**SaDSDE versus EFADE**	319	116	5.00E-05	Yes	Yes	425	40	6.47E-08	Yes	Yes
**SaDSDE versus MPEDE**	188	137	1.65E-03	Yes	Yes	336	99	3.37E-05	Yes	Yes
**SaDSDE versus EDEV**	189	136	1.34E-03	Yes	Yes	366	99	1.06E-05	Yes	Yes

From Tables 6 and 7, we can draw the following conclusions: (1) SaDSDE can obtain the global optimal solution on 10 unimodal functions (*f*_*1*_*-f*_*10*_) and 12 multimodal functions (*f*_*12*_*-f*_*16*_, *f*_*18*_, *f*_*20*_, *f*_*22*_*-f*_*24*_ and *f*_*25*_*-f*_*2*8_). (2) Except for *f*_*11*_, *f*_*17*_, *f*_*20*_*-f*_*21*_, *f*_*27*_ and *f*_*29*_-*f*_*30*_, the convergence precision of SaDSDE is best at *D* = 30 and *D* = 100, and SaDSDE is comparable to the other compared algorithms on *f*_*19*_, *f*_*22*_ *and f*_*28*_. In addition, at *D* = 30, Rcr-JADE is best on *f*_*17*_ and *f*_*29*_, EsDEr-NR is best on *f*_*11*_, *f*_*25*_ and *f*_*29*_, IMMSADE is best on *f*_*22*_, AGDE is best on *f*_*21*_, MPEDE is best on *f*_*17*,_ *f*_*20*_ and *f*_*29*_; at *D* = 100, EsDEr-NR is best on *f*_*11*_, IMMSADE is best on *f*_*17*_, MPEDE is best on *f*_*20*_*-f*_*21*_, *f*_*25*_ and *f*_*29*_, EDEV is best on *f*_*30*_. (3) The optimization performance of other algorithms decreases rapidly for the high dimensions, but SaDSDE still keep the excellent optimization performance.

Form the statistical histogram in Fig 4, SaDSDE and Rcr-JADE, EsDEr-NR, IMMSADE, AGDE, EFADE, MPEDE and EDEV can obtain the best results on 23, 6, 7, 6, 2, 1, 8 and 5 functions and obtain the second best results on 0, 1, 10, 2, 1, 4, 9 and 3 functions at *D* = 30, respectively. For the high-dimensional problem tests, the advantage of the proposed SaDSDE is more prominent. SaDSDE and 7 improved DE variants can obtain the best results on 23, 0, 1, 4, 0, 0, 5 and 1 functions and obtain the second best results on 2, 1, 2, 11, 1, 2, 9 and 2 functions at *D* = 100, respectively.

According to the statistical results in Fig 5, SaDSDE can obtain global optimums on 22 (= 10 unimodal cases+12 multimodal cases) functions in all dimensions, while 7 DE variants in turn obtain global optimums on 3 (= 1+2), 3 (= 1+2), 5 (= 1+4), 1 (= 1+0), 1 (= 1+0), 4 (= 1+3) and 4 (= 1+3) functions at *D* = 30. As the dimension increases, the number of global optimum decreases greatly for compared algorithms.

As shown in Fig 6, SaDSDE is significantly better than Rcr-JADE, EsDEr-NR, IMMSADE, AGDE, EFADE, MPEDE and EDEV on 19, 18, 19, 22, 21, 17 and 18 functions out of 30 functions at *D* = 30, respectively. On the contrary, SaDSDE is worse than 7 DE variants only on 6, 7, 2, 5, 6, 6 and 7 functions, respectively. At *D* = 100, SaDSDE is significantly better than all the competitors at least in 20 out of 30 functions and the advantage of proposed SaDSDE is more prominent.

According to Fig 7, SaDSDE is the best, and EsDEr-NR and MPEDE are respectively the second best at *D* = 30 and *D* = 100.

As shown in Table 8, we can observe that SaDSDE gets higher R+ values than R− values for all the compared DEs, and all the *p*-values are less than 0.05. It proves that SaDSDE outperforms other compared DE algorithms significantly.

#### B. Comparison with 3 non-DE algorithms

**Fig 8:**
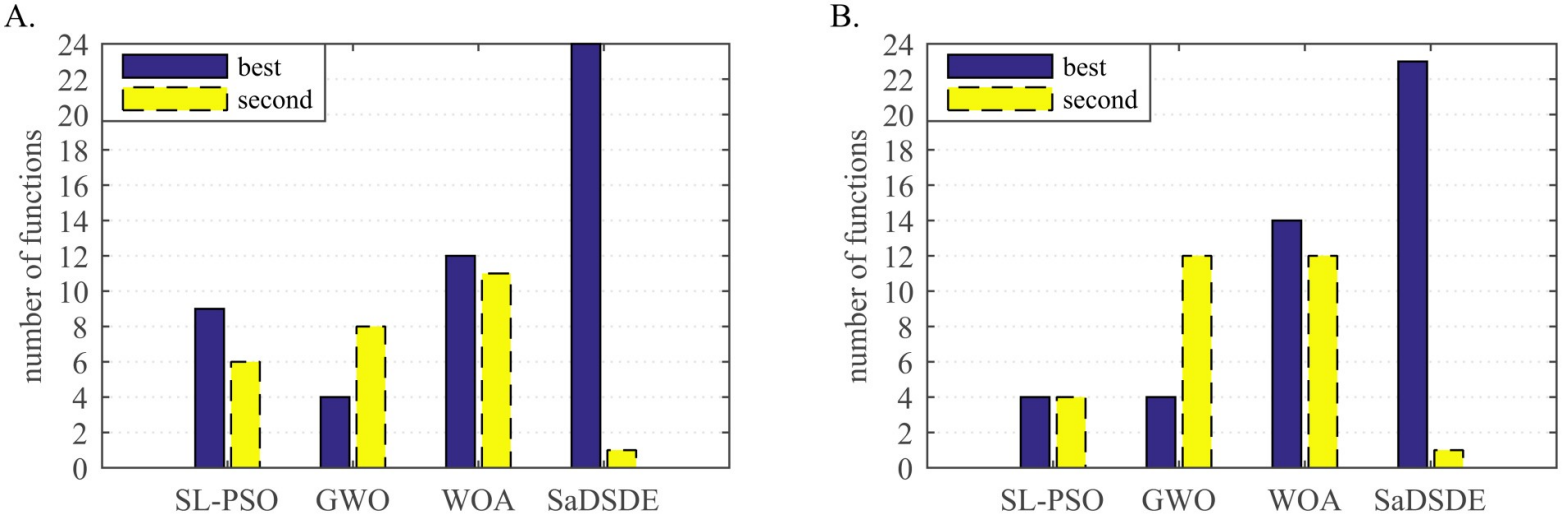
Number of cases on which each algorithm performs the best and second best in the comparison. (A) *D* = 30. (B) *D* = 100.

**Fig 9:**
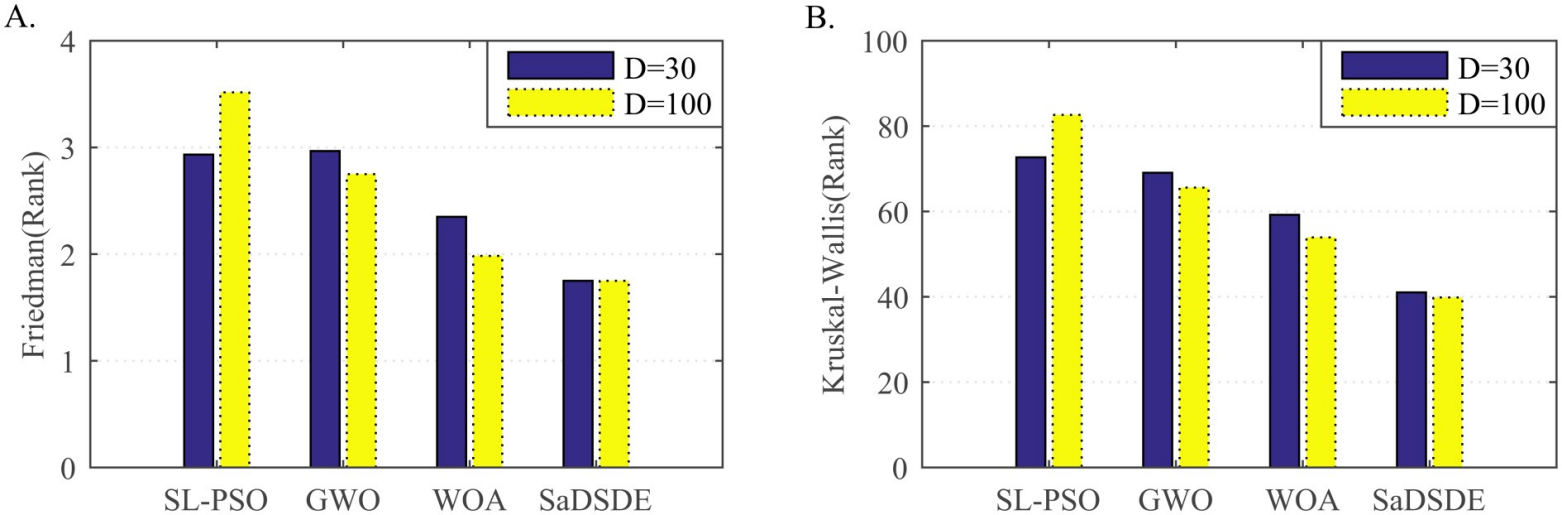
Non-parametric statistical test results over 30 independent runs. (A) Friedman test results. (B) Kruskal_Wallis test results.

DE simulates the natural evolution (such as mutation, crossover and selection) for global search. Unlike DE algorithm, SL-PSO [51], GWO [52] and WOA [53] are swarm intelligence optimization algorithms which simulate the social behavior of natural biological groups (such as foraging, nesting, migration, hunting, predation, etc.). SL-PSO, GWO and WOA are highly competitive swarm intelligence optimization algorithms. Therefore, in order to further verify the effectiveness of SaDSDE algorithm, SaDSDE is compared with SL-PSO, GWO and WOA algorithms. The obtained results (i.e., Mean and STD) and Wilcoxon signed-rank test results at the 0.05 significance level are shown in Tables 9 and 10. The number of the best and the second best results are shown in Fig 8. Additionally, the statistical analysis results for all test functions are listed in Fig 9 and the Wilcoxon rank-sum test results are represented in Table 11.

**Table 9:** The optimization results obtained by SaDSDE and 3 non-DE algorithms at *D* = 30.

*F*	SL-PSO	GWO	WOA	SaDSDE
	Mean ± STD	Mean ± STD	Mean ± STD	Mean ± STD
***f***_***1***_	2.83E-44±3.37E-44	2.34E-85±4.17E-85	*1*.*60E-191±0*.*00E+00*	**0.00E+00±0.00E+00**
***f***_***2***_	6.78E-02±4.83E-02	*9*.*46E-27±4*.*30E-26*	4.41E+03±3.15E+03	**0.00E+00±0.00E+00**
***f***_***3***_	1.76E-38±6.23E-38	1.10E-81±2.91E-81	*9*.*44E-186±0*.*00E+00*	**0.00E+00±0.00E+00**
***f***_***4***_	1.98E-23±1.12E-23	2.59E-49±2.33E-49	*2*.*22E-114±7*.*19E-114*	**0.00E+00±0.00E+00**
***f***_***5***_	4.36E-12±2.46E-12	*1*.*01E-21±1*.*41E-21*	6.46E-08±1.90E-07	**0.00E+00±0.00E+00**
***f***_***6***_	4.65E-45±7.14E-45	3.08E-86±9.99E-86	*2*.*07E-188±0*.*00E+00*	**0.00E+00±0.00E+00**
***f***_***7***_	3.63E-38±4.09E-38	2.29E-79±6.05E-79	*1*.*82E-184±0*.*00E+00*	**0.00E+00±0.00E+00**
***f***_***8***_	1.15E+02±3.95E+01	*8*.*78E-83±2*.*04E-82*	6.44E+01±6.70E+01	**0.00E+00±0.00E+00**
***f***_***9***_	5.79E-38±1.16E-37	8.18E-80±1.17E-79	*1*.*42E-184±0*.*00E+00*	**0.00E+00±0.00E+00**
***f***_***10***_	**0.00E+00±0.00E+00**	**0.00E+00±0.00E+00**	**0.00E+00±0.00E+00**	**0.00E+00±0.00E+00**
***f***_***11***_	1.25E+03±9.78E-06	*2*.*98E-04±1*.*58E-04*	**1.87E-05±2.12E-05**	1.25E+03±3.83E-06
***f***_***12***_	1.34E+01±3.19E+00	*1*.*47E-01±8*.*04E-01*	**0.00E+00±0.00E+00**	**0.00E+00±0.00E+00**
***f***_***13***_	*9*.*85E-04±3*.*21E-03*	1.45E-03±4.47E-03	3.20E-03±1.04E-02	**0.00E+00±0.00E+00**
***f***_***14***_	**0.00E+00±0.00E+00**	**0.00E+00±0.00E+00**	**0.00E+00±0.00E+00**	**0.00E+00±0.00E+00**
***f***_***15***_	4.02E-45±4.18E-45	1.86E-86±3.46E-86	*6*.*82E-194±0*.*00E+00*	**0.00E+00±0.00E+00**
***f***_***16***_	*6*.*16E-15±1*.*60E-15*	1.05E-14±2.87E-15	**0.00E+00±0.00E+00**	**0.00E+00±0.00E+00**
***f***_***17***_	4.84E+01±4.58E+01	2.62E+01±5.51E-01	*2*.*59E+01±2*.*21E-01*	**2.35E+01±6.98E-01**
***f***_***18***_	2.45E-02±2.39E-02	1.30E-22±1.07E-22	*2*.*18E-65±6*.*09E-65*	**0.00E+00±0.00E+00**
***f***_***19***_	**3.82E-04±0.00E+00**	**3.82E-04±7.40E-13**	**3.82E-04±0.00E+00**	**3.82E-04±0.00E+00**
***f***_***20***_	**2.19E-01±4.18E-02**	5.35E-01±7.87E-02	*3*.*68E-01±7*.*32E-02*	5.29E-01±8.87E-02
***f***_***21***_	*4*.*18E-01±7*.*51E-02*	4.31E-01±5.58E-02	**3.30E-01±7.09E-02**	5.00E-01±0.00E+00
***f***_***22***_	**0.00E+00±0.00E+00**	**0.00E+00±0.00E+00**	**0.00E+00±0.00E+00**	**0.00E+00±0.00E+00**
***f***_***23***_	**0.00E+00±0.00E+00**	*1*.*69E-01±4*.*27E-02*	**0.00E+00±0.00E+00**	**0.00E+00±0.00E+00**
***f***_***24***_	*3*.*77E-01±1*.*85E-01*	6.10E-01±3.57E-01	1.06E+00±1.39E+00	**0.00E+00±0.00E+00**
***f***_***25***_	*4*.*30E+00±2*.*05E+00*	8.17E+00±1.38E+00	**0.00E+00±0.00E+00**	1.25E+01±7.66E-01
***f***_***26***_	2.55E+01±8.28E+00	*1*.*90E+00±5*.*18E+00*	**0.00E+00±0.00E+00**	**0.00E+00±0.00E+00**
***f***_***27***_	*4*.*07E-17±1*.*55E-16*	5.24E-05±1.55E-04	3.63E-01±1.99E+00	**0.00E+00±0.00E+00**
***f***_***28***_	**0.00E+00±0.00E+00**	**0.00E+00±0.00E+00**	**0.00E+00±0.00E+00**	**0.00E+00±0.00E+00**
***f***_***29***_	**1.57E-32±5.57E-48**	8.40E-02±5.10E-02	1.91E-01±8.57E-01	*1*.*34E-10±2*.*75E-10*
***f***_***30***_	**1.10E-03±3.35E-03**	1.39E-01±1.39E-01	*5*.*26E-03±5*.*28E-03*	1.24E-01±3.61E-01
***+***	17	20	15	-
***-***	5	3	4	-
**≈**	8	7	11	-

**Table 10:** The optimization results obtained by SaDSDE and 3 non-DE algorithms at *D* = 100.

*F*	SL-PSO	GWO	WOA	SaDSDE
	Mean ± STD	Mean ± STD	Mean ± STD	Mean ± STD
***f***_***1***_	4.27E-15±2.74E-15	3.51E-41±3.48E-41	*3*.*69E-188±0*.*00E+00*	**0.00E+00±0.00E+00**
***f***_***2***_	1.06E+05±1.42E+04	*3*.*02E-03±9*.*79E-03*	4.62E+05±7.13E+04	**0.00E+00±0.00E+00**
***f***_***3***_	6.07E-07±2.65E-06	8.48E-38±8.31E-38	*2*.*09E-183±0*.*00E+00*	**0.00E+00±0.00E+00**
***f***_***4***_	3.15E-08±1.88E-08	1.18E-24±9.13E-25	*3*.*39E-112±1*.*58E-111*	**0.00E+00±0.00E+00**
***f***_***5***_	2.65E+00±7.10E-01	*2*.*04E-06±4*.*86E-06*	2.34E-02±8.78E-02	**0.00E+00±0.00E+00**
***f***_***6***_	1.82E-15±1.59E-15	1.38E-41±1.43E-41	*9*.*20E-191±0*.*00E+00*	**0.00E+00±0.00E+00**
***f***_***7***_	4.27E-09±2.45E-09	6.27E-35±1.04E-34	*2*.*52E-180±0*.*00E+00*	**0.00E+00±0.00E+00**
***f***_***8***_	1.60E+05±4.07E+05	1.*30E-40±1*.*53E-40*	1.27E+03±4.18E+02	**0.00E+00±0.00E+00**
***f***_***9***_	7.24E-08±3.75E-07	6.56E-35±6.19E-35	*4*.*07E-182±0*.*00E+00*	**0.00E+00±0.00E+00**
***f***_***10***_	2.00E-01±4.84E-01	**0.00E+00±0.00E+00**	**0.00E+00±0.00E+00**	**0.00E+00±0.00E+00**
***f***_***11***_	1.36E+04±1.05E-05	*8*.*48E-04±3*.*77E-04*	**2.50E-05±1.77E-05**	1.36E+04±5.13E-06
***f***_***12***_	6.84E+02±1.67E+02	**0.00E+00±0.00E+00**	**0.00E+00±0.00E+00**	**0.00E+00±0.00E+00**
***f***_***13***_	1.81E-03±4.34E-03	*1*.*03E-03±3*.*17E-03*	4.58E-03±1.45E-02	**0.00E+00±0.00E+00**
***f***_***14***_	**0.00E+00±0.00E+00**	**0.00E+00±0.00E+00**	**0.00E+00±0.00E+00**	**0.00E+00±0.00E+00**
***f***_***15***_	1.26E-15±7.25E-16	4.37E-42±4.41E-42	*9*.*55E-193±0*.*00E+00*	**0.00E+00±0.00E+00**
***f***_***16***_	1.04E-08±3.04E-09	*4*.*42E-14±4*.*03E-15*	**0.00E+00±0.00E+00**	**0.00E+00±0.00E+00**
***f***_***17***_	3.53E+02±3.71E+02	*9*.*68E+01±9*.*76E-01*	**9.65E+01±3.29E-01**	9.70E+01±6.77E-01
***f***_***18***_	2.57E+00±3.98E+00	3.82E-10±2.18E-10	*1*.*13E-64±5*.*78E-64*	**0.00E+00±0.00E+00**
***f***_***19***_	*1*.*23E+01±6*.*74E+01*	**1.27E-03±3.69E-12**	**1.27E-03±0.00E+00**	**1.27E-03±0.00E+00**
***f***_***20***_	*6*.*76E-01±5*.*70E-02*	8.58E-01±9.08E-02	**4.48E-01±1.23E-01**	9.31E-01±6.87E-02
***f***_***21***_	7.01E-01±3.18E-01	5.63E-01±9.11E-02	**4.43E-01±8.63E-02**	*5*.*00E-01±0*.*00E+00*
***f***_***22***_	5.59E-01±1.05E+00	*2*.*56E-14±1*.*56E-14*	**0.00E+00±0.00E+00**	**0.00E+00±0.00E+00**
***f***_***23***_	**0.00E+00±0.00E+00**	*5*.*05E-01±7*.*60E-02*	**0.00E+00±0.00E+00**	**0.00E+00±0.00E+00**
***f***_***24***_	2.27E+00±6.99E-01	1.17E+00±1.26E+00	*6*.*46E-01±2*.*03E+00*	**0.00E+00±0.00E+00**
***f***_***25***_	6.74E+01±3.63E+00	*4*.*05E+01±1*.*93E+00*	**0.00E+00±0.00E+00**	4.39E+01±7.11E-01
***f***_***26***_	8.34E+02±3.50E+01	*2*.*53E+00±4*.*40E+00*	**0.00E+00±0.00E+00**	**0.00E+00±0.00E+00**
***f***_***27***_	3.70E-08±2.22E-08	6.67E-05±1.80E-04	*1*.*35E-110±6*.*85E-110*	**0.00E+00±0.00E+00**
***f***_***28***_	*5*.*60E-01±6*.*60E-01*	**0.00E+00±0.00E+00**	**0.00E+00±0.00E+00**	**0.00E+00±0.00E+00**
***f***_***29***_	**1.56E-03±8.57E-03**	5.16E-01±7.38E-02	*2*.*81E-03±7*.*52E-04*	3.96E-02±2.27E-02
***f***_***30***_	**4.03E-03±8.89E-03**	5.09E+00±4.57E-01	*3*.*60E-02±1*.*69E-02*	9.33E+00±1.73E+00
***+***	23	19	12	-
***-***	3	4	7	-
**≈**	4	7	11	-

**Table 11:** The results of Wilcoxon rank-sum test over 30 independent runs.

Comparisons	*D* = 30					*D* = 100				
	R+	R-	*p*-value	*α* = 0.05	*α* = 0.1	R+	R-	*p*-value	*α* = 0.05	*α* = 0.1
**SaDSDE versus SL-PSO**	218	82	9.56E-04	Yes	Yes	358	48	3.13E-05	Yes	Yes
**SaDSDE versus GWO**	282	69	1.05E-03	Yes	Yes	239	112	1.79E-03	Yes	Yes
**SaDSDE versus WOA**	157	74	1.24E-02	Yes	Yes	122	109	2.28E-02	Yes	Yes

Analyzing the test results in Tables 9 and 10, SaDSDE is significantly better than SL-PSO, GWO and WOA on 17, 20 and 15 functions out of 30 functions at *D* = 30, respectively. At *D* = 100, SaDSDE performs better than 3 non-DE algorithms on 23, 19 and 12 functions. From the diagram in Fig 8, SaDSDE obtains best results on 24 and 23 functions at *D* = 30 and *D* = 100, respectively. Fig 9 shows that SaDSDE gets the best ranking, followed by WOA. SaDSDE is better than 3 non-DE variants significantly, as shown in Table 11.

#### 4.5.2 Convergence analysis

**Fig 10:**
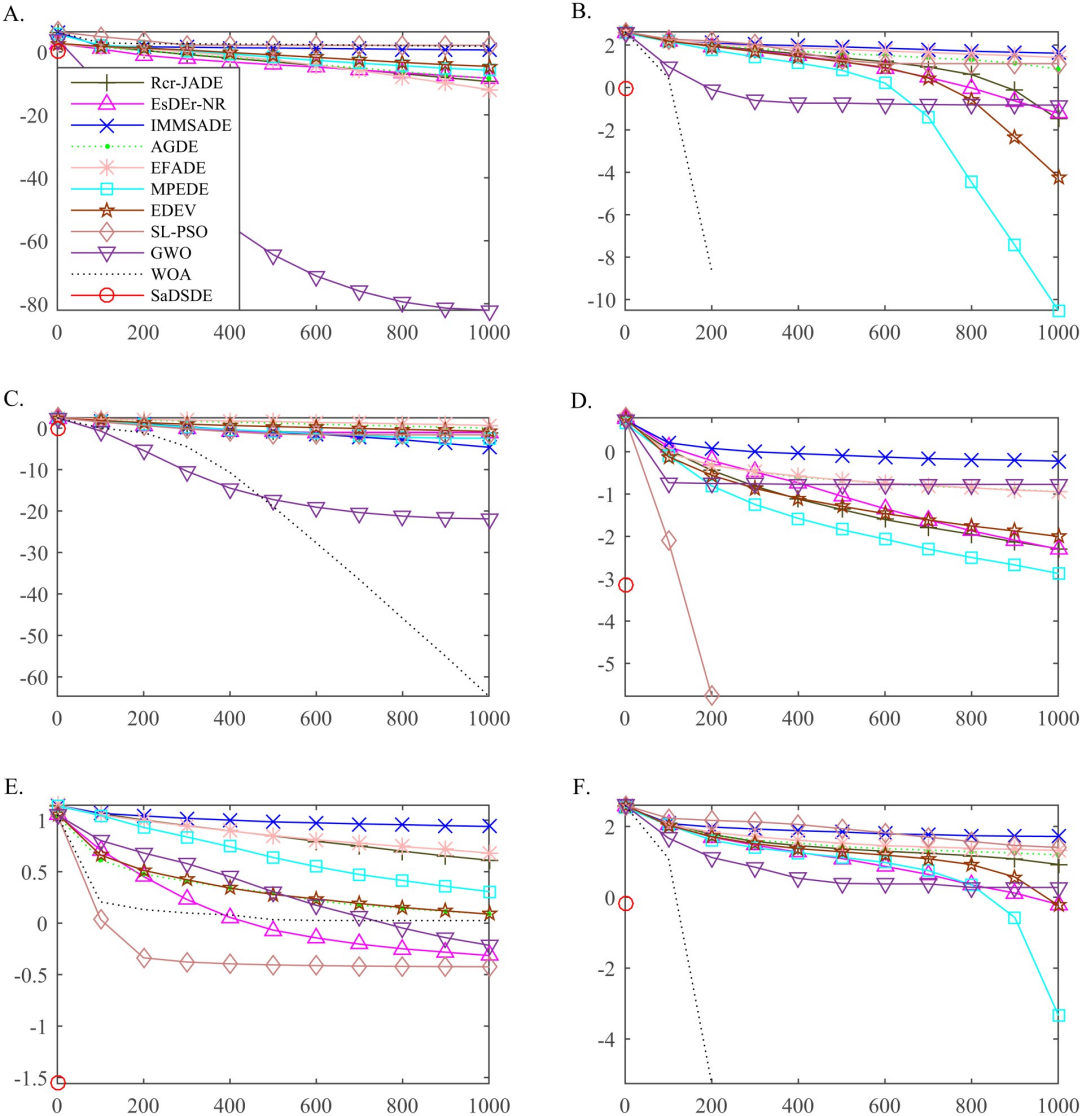
Convergence curves of the mean function error values for six test functions at *D* = 30. The horizontal axis and the vertical axis are generations and the mean function error values over 30 independent runs. The legends of Fig 10 (B-F) are the same as Fig 10(A). (A) *f*_*8*_. (B) *f*_*12*_. (C) *f*_*18*_. (D) *f*_*23*_. (E) *f*_*24*_. (F) *f*_*26*._

**Fig 11:**
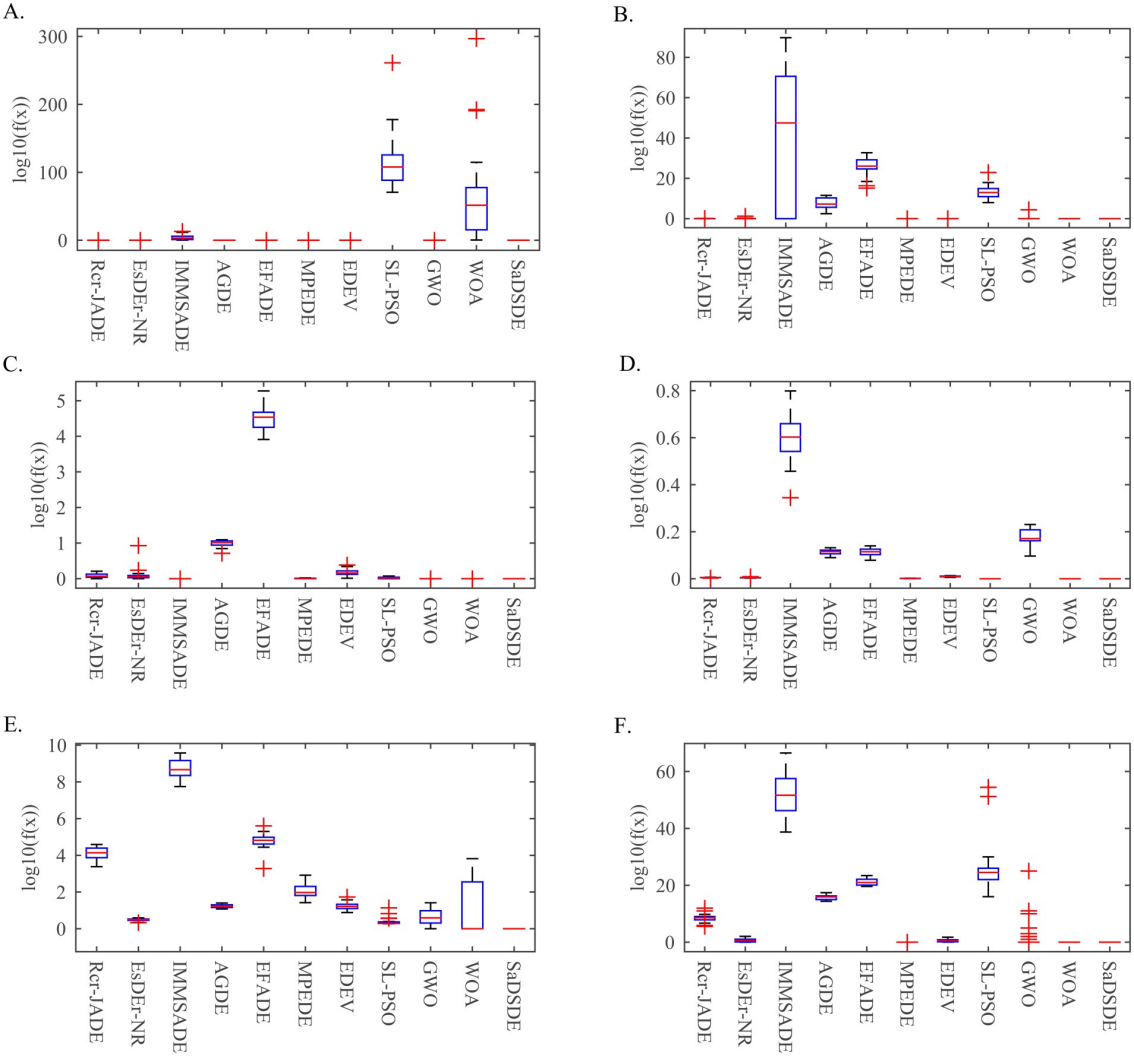
Box plots of the result of solution error at *D* = 30 over independent 30 runs. (A) *f*_*8*_. (B) *f*_*12*_. (C) *f*_*18*_. (D) *f*_*23*_. (E) *f*_*24*_. (F) *f*_*26*_.

In order to study the evolution properties, convergence curves are utilized to prove the difference, as illustrated in Fig 10. Furthermore, to illustrate the distribution of the results of each algorithm, the box plots of function solutions of all the algorithms on 6 functions (1 unimodal function and 5 multimodal functions) at *D* = 30 are depicted in Fig 11.

Fig 10 shows that SaDSDE can obtain the global optimum and has the best convergence rate on 6 functions in the minimum generations, while other compared algorithms have strapped into "evolution stagnation" on different functions. For example, Non-Continuous Rastrigin’s Function (*f*_*26*_) is multi-modal, non-separable and asymmetrical function and the local optima’s number is huge, DE is easy to suffer premature convergence to be trapped in one of its many local minima. SaDSDE can find the global optimal solution. However, IMMSADE, EFADE, AGDE and GWO have trapped into “evolution stagnation”. In addition, we can see that SaDSDE performs more consistently than other algorithms on these problems, as shown in Fig 11.

As evident from above analysis, the proposed SaDSDE demonstrates the better performance of convergence precision and robustness than most of the compared algorithms. To summarize, the proposed SaDSDE has the best convergence precision and stability. The reason is that the proposed mutation strategy, scaling factor self-adaption strategy and dynamic adjustment strategy of the exploration ability control factor achieve a better balance of exploration and exploitation.

### 4.6 Efficiency analysis of proposed algorithmic components

The proposed algorithm represents a combined effect. Therefore, we do the efficiency analysis of proposed algorithmic components, including dual-strategy mutation operator, scaling factor self-adaption strategy and dynamic adjustment strategy of exploration ability control factor. Some variants of SaDSDE are listed as follows.

1. To study the effectiveness of scaling factor self-adaptation strategy, SaDSDE variants adopt dynamic *λ* and fixed scaling factor of *F* = 0.3, *F* = 0.7 and random real number in [0, 1], which are respectively named SaDSDE-1, SaDSDE-2 and SaDSDE-3 one by one.
2. To verify the contribution of dynamic adjustment strategy of the exploration ability control factor (*λ*), SaDSDE variants with self-adaptive *F*, *λ* = 0.3, *λ* = 0.7 and random real number in [0, 1] are respectively named SaDSDE-4, SaDSDE-5 and SaDSDE-6 for short.

**Fig 12:**
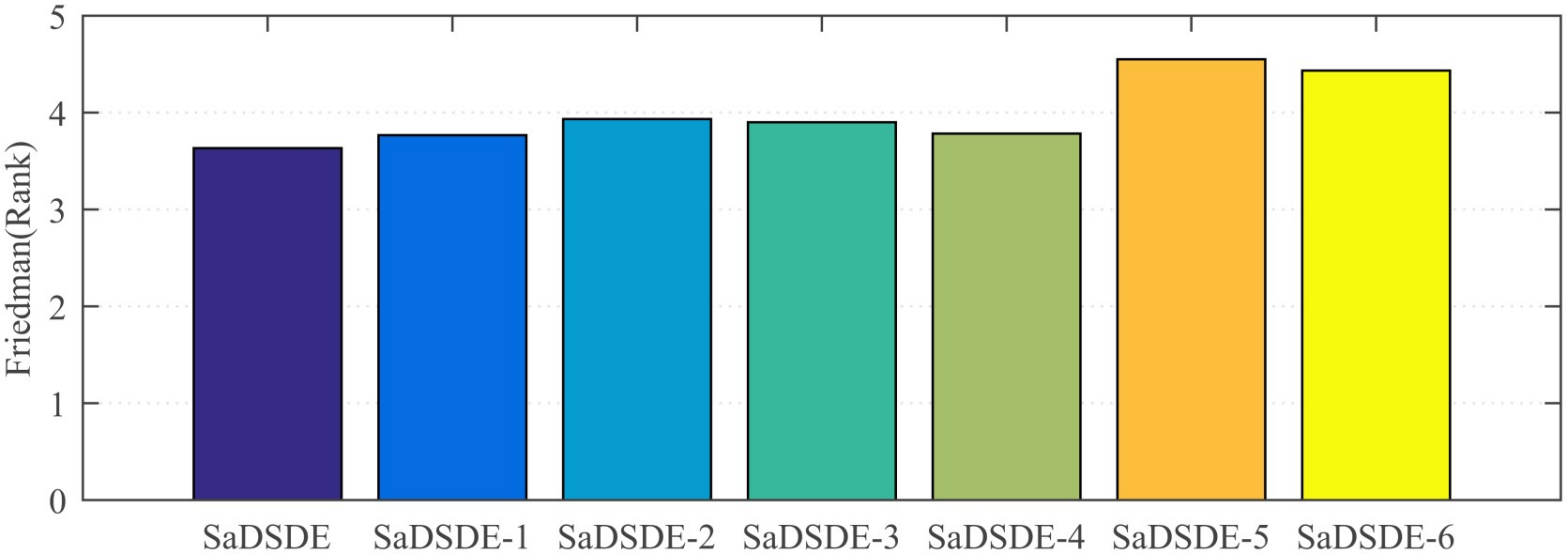
The Friedman test results over 30 independent runs.

In order to evaluate and compare the performance of different SaDSDE variants, Friedman test and Wilcoxon’s rank-sum test are employed, and the test results are plotted in Fig 12 and Table 12, respectively. Observing the test results in Fig 12 and Table 12, we can obtain the following conclusions easily. (1) From Fig 12, the proposed SaDSDE and SaDSDE-1 are respectively the best and the second best, followed by SaDSDE-4, SaDSDE-3, SaDSDE-2, SaDSDE-6 and SaDSDE-5. It is clear that the combined effect of proposed algorithm components is best. (2) From Table 12, there is no significant performance difference between proposed SaDSDE and SaDSDE variants with exploration ability control factor, while proposed SaDSDE is better than SaDSDE-5 with a larger exploration ability control factor significantly. The validity of two proposed strategies is verified by means of above experimental comparisons. It is note that the contribution of dynamic adjustment strategy of the exploration ability control factor is larger than scaling factor self-adaptation strategy. In other words, although the scaling factor self-adaption is effective, SaDSDE is less susceptible to scaling factor.

**Table 12:** The results of Wilcoxon’s rank-sum test of proposed SaDSDE and 6 SaDSDE variants over 30 independent runs.

Comparisons	R+	R-	*p*-value	*α* = 0.05	*α* = 0.1
**SaDSDE versus SaDSDE-1**	14	7	9.92E-01	No	No
**SaDSDE versus SaDSDE-2**	16	5	9.02E-01	No	No
**SaDSDE versus SaDSDE-3**	15	6	9.32E-01	No	No
**SaDSDE versus SaDSDE-4**	24	12	6.03E-01	No	No
**SaDSDE versus SaDSDE-5**	114	39	2.85E-02	Yes	Yes
**SaDSDE versus SaDSDE-6**	55	11	2.75E-01	No	No

### 4.7 Adaptability analysis to high-dimensional problems

**Fig 13:**
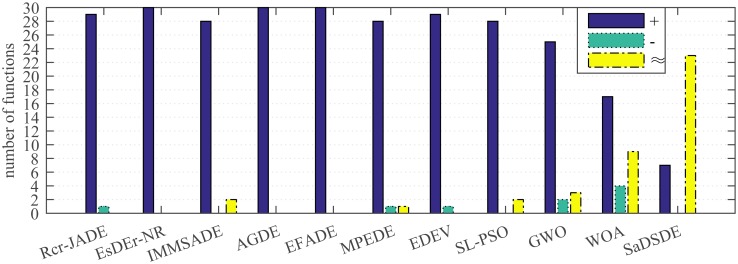
The Wilcoxon’s signed-rank test results with a significance level of 0.05 over 30 independent runs. Signs“+”, “-” and “≈” indicate the number of functions that the performance of algorithm for low dimensional problems is better than, worse than and similar to the performance for high dimensional problems, respectively.

In order to evaluate the adaptability to high-dimensional problems of each algorithm, we conduct Wilcoxon’s signed-rank test and Wilcoxon rank-sum test at the 0.05 significance level for each algorithm between its own 30 variables and 100 variables. The nonparametric statistical test results are shown in Fig 13 and Table 13. From Fig 13, we can obtain the following observations: (1) For EsDEr-NR, AGDE and EFADE, the low-dimensional performance is significantly better than high-dimensional performance on all the test functions. (2) For SaDSDE, the low dimensional performance is significantly better than, worse than and similar to high dimensional performance on 7 and 23 out of 30 functions, respectively. (3) For IMMSADE, GWO, WOA and SaDSDE, the difference is not statistically significant in Table 13, while the gap between R^+^ and R^-^ of SaDSDE is significantly smaller than IMMSADE, GWO and WOA. Therefore, SaDSDE has the best adaptability to high-dimensional problems.

**Table 13:** The results of Wilcoxon rank-sum test over 30 independent runs.

Comparisons	R+	R-	*p*-value	*α* = 0.05	*α* = 0.1
**Rcr-JADE at *D* = 30 versus Rcr-JADE at *D* = 100**	455	10	7.29E-04	Yes	Yes
**EsDEr-NR at *D* = 30 versus EsDEr-NR at *D* = 100**	465	0	2.88E-06	Yes	Yes
**IMMSADE at *D* = 30 versus IMMSADE at *D* = 100**	406	0	1.19E-01	No	No
**AGDE at *D* = 30 versus AGDE at *D* = 100**	465	0	6.74E-06	Yes	Yes
**EFADE at *D* = 30 versus EFADE at *D* = 100**	465	0	7.60E-07	Yes	Yes
**MPEDE at *D* = 30 versus MPEDE at *D* = 100**	426	9	5.54E-04	Yes	Yes
**EDEV at *D* = 30 versus EDEV at *D* = 100**	454	11	2.83E-04	Yes	Yes
**SL-PSO at *D* = 30 versus SL-PSO at *D* = 100**	406	0	1.27E-02	Yes	Yes
**GWO at *D* = 30 versus GWO at *D* = 100**	345	33	3.87E-01	No	No
**WOA at *D* = 30 versus WOA at *D* = 100**	178	53	9.16E-01	No	No
**SaDSDE at *D* = 30 versus SaDSDE at *D* = 100**	28	0	8.94E-01	No	No

### 4.8 Discussions on the comparison results

A series of experiment comparisons have proved the effectiveness of SaDSDE. The reasons can be concluded as follows. (1) The novel dual-strategy mutation operator gives attention to exploitation and exploration ability simultaneously. Exploration can make the algorithm search every promising solution area with good diversity, while exploitation can make the algorithm execute a local search in some promising solution areas to find the optimal point with a high convergence rate. (2) In scaling factor self-adaption strategy, the scaling factors of each individual are set for individuals to make full use of the differences in their fitness. Therefore, the superior individuals are assigned relatively smaller values to exploit their neighborhoods in which better solutions. On the contrary, the inferior individuals are assigned larger values to explore further areas in the solution space. (3) The exploration ability control factor is adjusted dynamically according to the generations, which takes into account the evolutionary property in different evolution stages. It can get a good balance between exploration and exploitation ability to make the algorithm search promising solution area and help algorithm jump over the trap of local optimal solution.

## Conclusions

The novel mutation operator with “DE/best/2” operator and “DE/rand/2” operator is proposed in this paper. The “DE/rand/2” mutation operator can expand the search area in the later stage of evolution and avoid suffering premature convergence. The “DE/best/2” mutation operator can accelerate the convergence speed greatly. The performance of the algorithm is excellent even if *CR* is set to a fixed value. Experiment results show that: (1) SaDSDE is not sensitive to the population size and it is easier to implement. (2) SaDSDE has the best performance among all the compared variants. (3) For the high-dimensional functions, with the same-scale population and iterations, SaDSDE can still get the excellent global optimization performance, while there is serious performance degradation for other compared algorithms. In other words, SaDSDE has the best adaptability to high-dimensional problems.

As a continuation of this research, we will focus on multi-objective optimization (MOO) problems and some actual engineering applications, such as flight conflict resolution and image registration, etc.
